# Simulation of microarray data with realistic characteristics

**DOI:** 10.1186/1471-2105-7-349

**Published:** 2006-07-18

**Authors:** Matti Nykter, Tommi Aho, Miika Ahdesmäki, Pekka Ruusuvuori, Antti Lehmussola, Olli Yli-Harja

**Affiliations:** 1 Institute of Signal Processing, Tampere University of Technology, Tampere, Finland

## Abstract

**Background:**

Microarray technologies have become common tools in biological research. As a result, a need for effective computational methods for data analysis has emerged. Numerous different algorithms have been proposed for analyzing the data. However, an objective evaluation of the proposed algorithms is not possible due to the lack of biological ground truth information. To overcome this fundamental problem, the use of simulated microarray data for algorithm validation has been proposed.

**Results:**

We present a microarray simulation model which can be used to validate different kinds of data analysis algorithms. The proposed model is unique in the sense that it includes all the steps that affect the quality of real microarray data. These steps include the simulation of biological ground truth data, applying biological and measurement technology specific error models, and finally simulating the microarray slide manufacturing and hybridization. After all these steps are taken into account, the simulated data has realistic biological and statistical characteristics. The applicability of the proposed model is demonstrated by several examples.

**Conclusion:**

The proposed microarray simulation model is modular and can be used in different kinds of applications. It includes several error models that have been proposed earlier and it can be used with different types of input data. The model can be used to simulate both spotted two-channel and oligonucleotide based single-channel microarrays. All this makes the model a valuable tool for example in validation of data analysis algorithms.

## Background

The emergence of several high throughput measurement technologies provides new possibilities to study biological organisms at the system level. New technologies produce such large amounts of data that can no longer be analyzed by hand. This has made computational techniques an inseparable part of data analysis. Although new computational methods are continuously proposed for data analysis, their performance can not be objectively evaluated. This remains as a fundamental problem in method development. Typically validation of data analysis methods is based on clinically determined labels of biological samples. If the computational method produces results which are consistent with the predetermined labels, then the method is considered to work reliably. This approach, however, relies entirely on *a priori *information about the data. Furthermore, the clinical classification of samples is not always unambiguous [1,2].

A more objective approach to validate the data analysis methods is to use data whose characteristics and ground truth are known [3,4]. Unfortunately, in real life problems this kind of data usually does not exist. Thus, to obtain data with known ground truth, one needs to produce the data by simulation. If simulated data is used to evaluate the performance of the analysis methods, can it be guaranteed that the same performance is obtained with real data also? To get meaningful results, the simulated data and the real biological data have to have similar biological and statistical characteristics.

A problem in the validation of data analysis algorithms using simulated data is that there is always an underlying mathematical model that is used to simulate the data. Thus, when different computational methods are compared, this approach favors the ones that implement the same assumptions as the data generation process does. While this is a fundamental problem, it can be circumvented by evaluating the methods using simulated data produced by different kinds of models. When the results are combined, the bias due to the model assumptions can be avoided.

Numerous studies have focused on mathematical modeling of biological and measurement errors, including both stochastic noise and systemic bias [5-11]. These studies have improved the analysis methods by utilizing the knowledge about the data properties [7]. This knowledge can be utilized in the generation of simulated data as well.

The error model itself is not enough for the simulation of biologically and statistically accurate data. Before an error model can be applied, the ground truth biological signal needs to be obtained. Depending on the application, a biological signal can be obtained for example by sampling a proper distribution or by modeling and simulating the biological system using differential equation models [12].

Once the biological ground truth signal has been generated and the error model has been applied, simulated data is still not comparable to real measurement data. Real data is always extracted from a measurement system. In the case of gene expression microarrays, image processing algorithms are used to read the spot values from the scanned slide image. The applied grid alignment, segmentation and data extraction algorithms have a significant effect to the obtained data [13].

There are numerous possible applications for a simulation model that can simulate realistic biological measurement data. The most obvious application is the validation and improvement of data analysis algorithms [3,4,14]. In addition, different data extraction algorithms can effectively be tested under different noise conditions. If the biological ground truth model is accurate enough one might even be able to simulate entire microarray experiments. If this could be done before performing expensive laboratory experiments, the proposed hypotheses could be tested with simulated data. This could help in finding problems in the design of the experiment and, thus, potentially save significant amount of time and money. 

While all the steps of the simulation process have been extensively studied separately [6,7,15-18], not much work has been done to combine all the steps. We propose a model that combines these steps and can be used to produce microarray data with realistic biological and statistical characteristics. The proposed model is modular and it can be easily extended to include new error models and even new measurement technologies. The current implementation supports the simulation of spotted two-channel microarrays and oligonucleotide based single-channel microarrays. We have implemented the model in Matlab environment [see additional file 1]. The simulation model is also available for download at our companion web page [19]. 

Biologically meaningful input data can be obtained from various sources. We introduce some possibilities how this data can be obtained. We then review several previously published error models which model biological and measurement technology specific errors, and which can be used to add realistic statistical properties to the simulated data. The result data is used as a basis for simulating the production of the microarray slides. After that, we discuss about the final step in obtaining realistic measurement data: the extraction of the gene expressions from the slide. Finally we demonstrate the applications of the proposed model by examples.

## Generation of the ground truth data

Depending on the application, the requirements for the ground truth data may vary. A typical microarray experiment includes comparison of different classes of samples, measuring a response to a perturbation, or measuring time series behavior. Validation of the data analysis methods developed for each of these applications sets different requirements for the ground truth data.

The simplest approach to generate the ground truth data is to sample data randomly from a specific distribution. First the distribution and its parameters can be estimated from real measurements. Next the ground truth data can be obtained by sampling a simulated ideal distribution with estimated parameters [7,15]. This approach can be adequate for several applications. The detection of differentially expressed genes is often based on the comparison of statistical properties of microarray data from two different samples, for example from two different cancer types. Therefore, the ground truth data suitable for validating data analysis methods can be obtained simply by sampling two distributions with different parameters.

If purpose of the data analysis is to study the behavior of the system in more detail, for example to study responses to perturbations, then biologically more detailed data can be generated. Because microarray technology measures gene expressions, the natural source for biological data would be a model of a genetic regulatory network (GRN). Unfortunately, GRNs are not generally known well enough so that they could be utilized in data simulation [20].

However, in some cases parts of the networks are known and even simulation models that include parts of the genetic regulatory mechanisms have been proposed [12,17]. These kinds of models would be ideal for the generation of ground truth data. If a model is accurate enough, even hypotheses about the behavior of the real system could be tested before a real microarray experiment is done.

Generation of data with biologically meaningful characteristics does not require the modeling of real GRNs [18]. Instead one can use networks with random topology. If the interactions between network components are modeled properly, for example by utilizing interaction information from real GRNs, one could produce data with realistic characteristics [20].

Once the network model has been obtained and mathematical models for interactions have been formulated, the expression values of the genes in the network need to be simulated. There are several publicly available software packages that can be used to accomplish this task [21,22].

Yet another application for microarray data is network inference, that is, learning the network structure and the interaction rules between the network components from time series or perturbation measurements.

In network inference, the modeling of control mechanisms of a network plays an essential role. Therefore, it is not necessary that all the interactions correspond to the ones of a real network and thus, even coarse scale models can be used. For example, it is shown that very simple models, even random Boolean networks, can capture some of the essential characteristics of real GRNs [23,24]. Thus it may be sufficient to use for example a Boolean network as a ground truth in network inference studies [25].

Real measurement data can also be used as ground truth data. This is the case, for example, if we want to study how our data analysis algorithm performs under different types and amounts of noise. By adding noise to real measurement data we can effectively test if the performance of our data analysis algorithms degrades as the amount of noise increases. This can give us valuable insight into the robustness of the algorithms.

## Microarray simulation model

In this section, a model for microarray measurements is presented. The model can use input data from numerous different sources. In practice, there is no limitation on what kind of simulator or software is used to generate the ground truth data.

**Figure 1:**
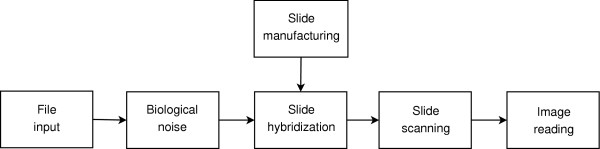
**Microarray simulation model**. Block diagram of the microarray simulation model.

The proposed simulation model is modular and the configuration is very flexible. The structure of the model is presented in Figure 1. Each module is independent of the others, and can easily be replaced.  This, for example, makes it possible to easily change the error model for the biological noise.

In the following we will discuss the most important characteristics of each module. Model parameters are listed in Tables 1, 2, 3, 4, 5. A more detailed documentation about the effect and usage of each parameter can be found on our companion web page [19].

### File input

Input data to the model is read using a file input module. This module converts the data to the internal format of the simulation model. Input data can be gene expression values or expression ratios. For example data from Affymetrix .cel files or simulated expression values can be used. In addition to data itself, the user should specify spot locations on the slide and their identifiers, such as probe names. Requirements for the input data are listed in Table 6. More detailed information about the format of the input data are given on the companion web page [19].

### Biological and measurement noise

The most important part in the simulation of realistic microarray data is the modeling of biological and measurement technology specific errors because they define the statistical characteristics of the simulated data. Biological errors are typically considered to include the internal stochastic noise of the cells and error sources related to sample preparation [16,26]. This type of intrinsic noise is present in all measurements, regardless of the measurement technology. Measurement errors, on the other hand, include error sources that are directly related to the measurement technology and its limitations, for example bias due to the used dyes. The properties of this kind of extrinsic noise depend on the measurement technology [5]. In addition to the fact that the simulated ground truth data is measurement error free, there is another major difference compared to real microarray data. Microarray data are usually measurements from cell populations. Thus the measured values are average expression values of all the cells in the population while the simulated data essentially presents the behavior of a single cell. Furthermore, it is difficult to prepare a sample containing only one type of cells. Therefore, the measured data is typically from a heterogeneous cell population, for example from a mixture of different types of cells [27]. The simulated data can be made more realistic by introducing a population effect. This can be done by using a kernel function to spread the ideal expression patterns as proposed in [28]. The population effect blurs the simulated ground truth data so that all the details can not be observed. Small variations occurring only in some cells can not be observed because they are covered by the large trends of the majority of the cells.

After the population effect has been taken into account, we can add biological and measurement errors to the simulated data. There have been numerous studies characterizing the properties of the error sources [5-11]. While the formulations of different error models are slightly different, the main components in all the models are the same. All of these models contain components that are dependent and components that are independent of the expression level. Thus, the errors are considered to be nonlinear in nature. Biological and measurement errors can be presented in the compact form

*y *= *f *(*x*) + *e*,       (1)

where *f *is a nonlinear function, depending on the gene expression level *x*, *e *is an error term independent of gene expression level, and *y *is the observed expression value. Function *f *includes all error sources that are dependent on the true underlying biological gene expression level *x*. Thus, error term *e *and function *f *include both stochastic noise and systemic bias that originate from biological and measurement technology specific error sources.

To make it possible to estimate the parameters of the error models from real data, error terms are usually factorized into a more detailed form. Typically an error model includes separate terms for gene specific noise, measurement specific noise, array specific noise, biological sample specific noise, noise independent of all these, and so on [6,7]. Some of the components model the intrinsic noise, that is, errors from biological origin while other components represent the extrinsic noise, that is, errors from the microarray measurement technology. However, usually both of these error types are modeled together regardless of their origin.

As there are error sources that are gene, array and biological sample specific, there needs to be a way to implement all these in the model. In addition to these error sources, there may be technology specific details which have to be considered. Affymetrix type oligonucleotide arrays contain several probes that are a part of the same probe set and thus measure the same gene. Furthermore, perfect match (PM) and mismatch (MM) probes need to be handled independently in the error model [11]. These issues are taken into account in the simulation model design, and all these type of errors can easily be included. For details on how different types of error sources can be implemented, see the documentation available on the companion web page.

Our microarray simulation model includes several error models proposed in the literature [6-11]. Along with the models, methods for estimating model parameters from real measurement data have been proposed [7,9,11]. These methods can be used to estimate realistic parameters for the simulation. Some of the implemented error models are for oligonucleotide and some for cDNA data. Thus, to get statistically accurate results the right type of error model needs to be used together with the proper array type. The error models and their parameters are summarized in Table 5. After the error model and the population effect have been applied, the simulated data has realistic biological and statistical characteristics.

### Slide manufacturing

To model a real microarray experiment it is not enough to simulate the gene expressions and to apply the error model, but the extraction of the data from slides has to be considered too [13]. Thus we need to model the microarray manufacturing process.

**Figure 2:**
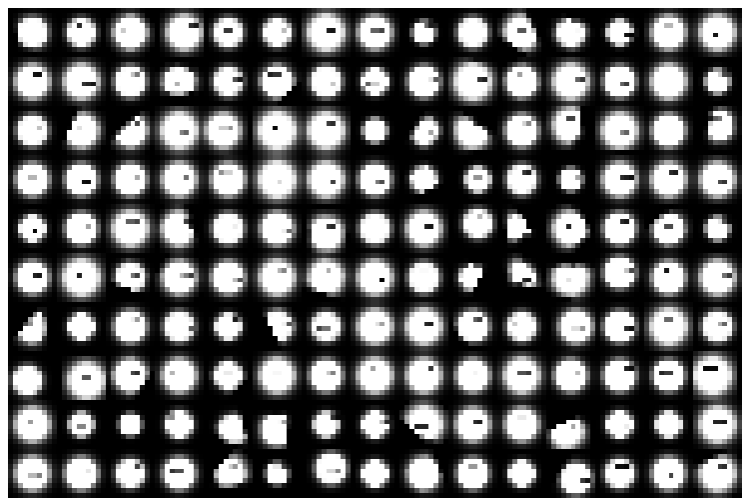
**Slide generation errors**. Errors in slide image generation are demonstrated. There is large variation in the spot size. In addition many spots have unideal shapes.

**Figure 3:**
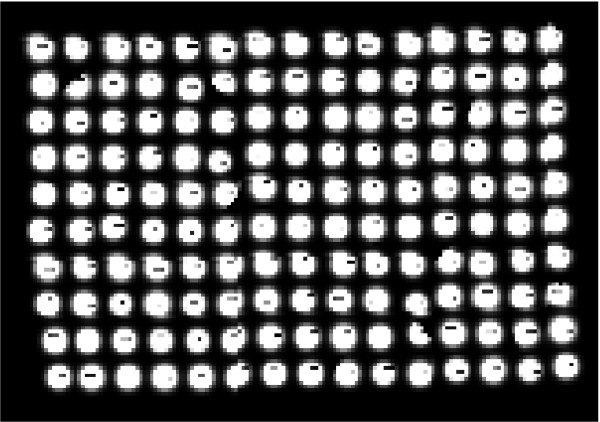
**Drift in spot alignment**. Systematic drift due to the unideal printing of the microarray slide can be introduced with used adjustable parameters.

A slide image is simulated using a user specified layout, that is, how many subarrays there are on the slide and how many spots or probes are in each row and column. Slide simulation introduces several error sources that are often visible in real microarray slide images. These include variation in the spot position and size. In addition the marks done by a print tip and deformations in the spot shape can be produced. For example, one type of deformations are chords that are cut away from the spots. These error sources are demonstrated in Figure 2. It is also possible to make the subarray layout imperfect by applying a non-linear error which makes the subarrays to drift from their ideal rectangular layout. This is shown in Figure 3. All the error sources can be controlled probabilistically by user adjustable parameters (see Table 2).

### Slide hybridization

**Figure 4:**
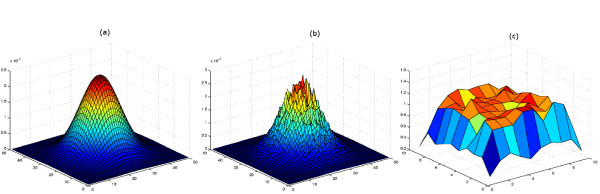
**Simulated spots**. Shape of (a) simulated noise free cDNA spot, (b) noisy cDNA spot, and (c) noisy single-channel oligonucleotide array spot. Intensity of the spot is determined by the corresponding expression value.

The slide hybridization step simulates the shape of the hybridized spot on the microarray. Several models for spot shape have been proposed. As different array technologies produce different types of spots, there is no single spot model that is suitable for all types of arrays. For example, it is shown that Gaussian distribution can be successfully fitted over cDNA microarray spots [29]. Recent studies have also introduced more detailed spot models [30]. We have implemented several models for the spot shape, including Gaussian and polynomial-hyperbolic spot shapes [30]. Parameters for the spot shapes can be set by the user. The ideal shape of the spot is corrupted by multiplicative Gaussian noise, again with user specified parameter values for the noise. The hybridized spot is then obtained by multiplying the noisy spot shape by the corresponding expression value. Spot generation with the Gaussian spot shape is presented in Figure 4(a–b). In the case of a single channel oligonucleotide microarray, rectangular spot corrupted by additive Gaussian noise is used. An example of a simulated oligonucleotide microarray spot is shown in Figure 4(c).

**Figure 5:**
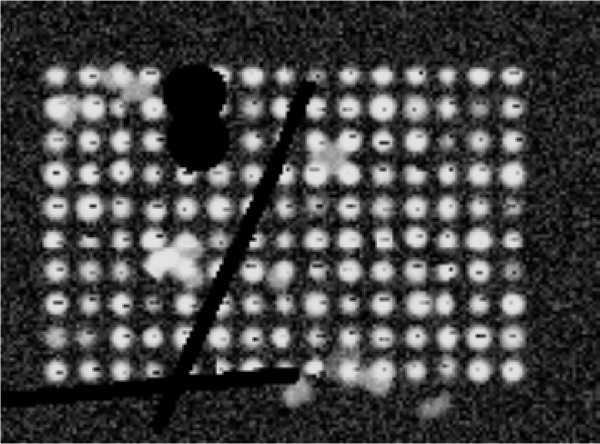
**Hybridization errors**. Hybridization errors are demonstrated. Spot bleeding, scratches, air bubbles and background noise are clearly visible.

Like previously in the slide generation phase, the user can introduce several hybridization errors that are typical for microarrays. Errors include background noise, spot bleeding, scratches, and air bubbles. These are demonstrated in Figure 5.

While the most relevant of these errors may depend on the array type, the simulation model makes it possible to use the same error sources on both spotted two-channel and oligonucleotide based single-channel arrays. Introduction of these types of error sources might be of interest in validation of grid alignment and segmentation algorithms.

### Slide scanning

In real experiments the hybridized slide is digitized by scanning. As a result a digital RGB image is obtained in which each color channel corresponds to the intensity information from different dyes. While the modern scanners are usually of high quality, they still have an effect on the obtained data, for example, in the form of the dynamic range. All scanners have a finite dynamic range, and thus some measurement values might saturate.

The scanner can also be a source for other type of errors. Because the slide is read by scanning each dye color separately, it might be possible that channels do not align perfectly. Furthermore, it is not guaranteed that the slide is always scanned exactly straight. All these types of errors are included into the model.

### Image reading

The final step in obtaining the realistic simulation data is to extract the expression values from the image. Because our simulation model produces images similar to real microarray slides, one can conveniently use any microarray feature extraction software.

We have however included an automatic grid alignment and image segmentation algorithm into the simulation model so that the data can be automatically extracted from images. These default algorithms can be easily replaced by other extraction algorithms.

## Results and discussion

**Figure 6:**
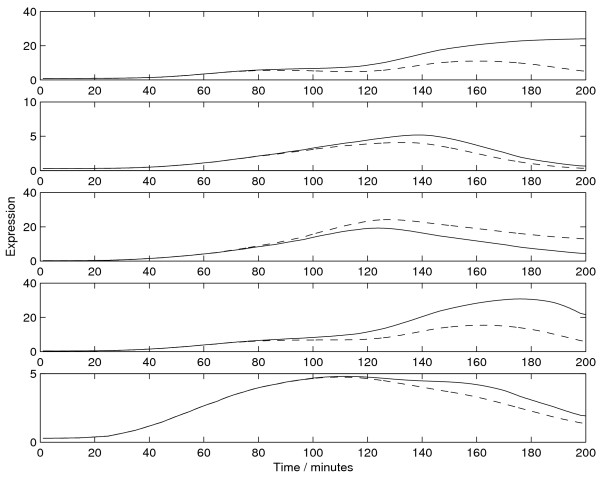
**Simulated ground truth signals**. Gene expression profiles of the selected genes. The effect of the gene knockout to the expression profiles is clearly observable. Reference signal is shown with solid and test signal with dashed line.

**Figure 7:**
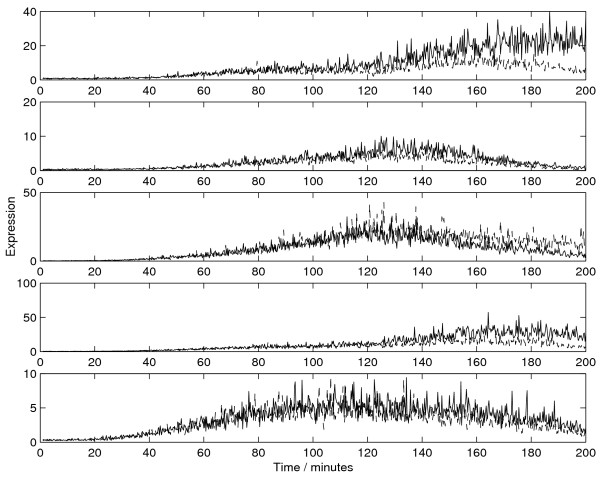
**Simulated ground truth signals with noise added**. Gene expression profiles of the same genes as in Figure 6 with measurement noise added. Small trends (for example lowest sub figure) in the signals are covered by noise. Reference signal is shown with solid and test signal with dashed line.

We first demonstrate the use of the proposed microarray model using simulated gene expression data. The ground truth biological signals are generated using random network topology with kinetic rate laws that present rates for transcription processes, and kinetic rate laws for degradation rates of the gene products [18]. The details about the data generation can be found on our companion web site. We use a gene knock out experiment as a case study [31]. The reference data is obtained by simulating the generated network. Then the test sample is obtained by knocking out a randomly chosen gene from the network and then running the same simulation using the network with the knocked out gene. Simulated gene expression profiles of a few selected genes that were affected by the knock out are shown in Figure 6. Next an error model is applied to the obtained ground truth data. We use the hierarchical error model to model the biological and the measurement specific noise [6]. Figure 7 illustrates the simulated gene expressions profiles after adding the noise.

**Figure 8:**
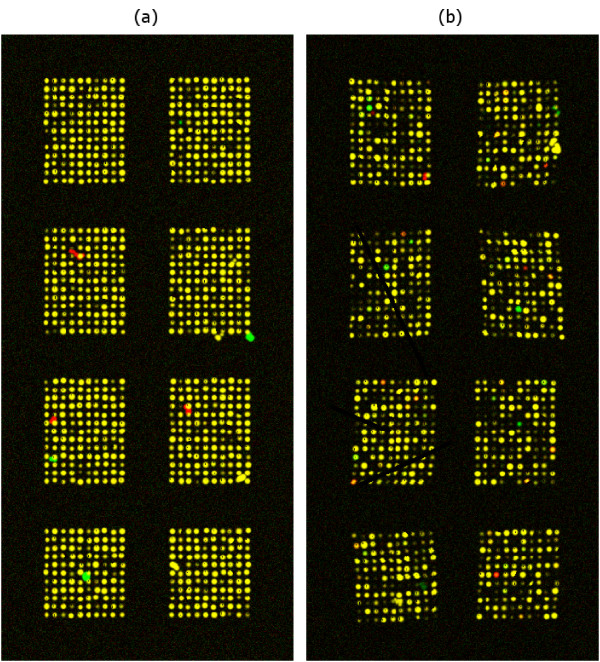
**Simulated spotted microarray slide images**. Simulated slide images at time instants (a) 10 and (b) 200 minutes. Several error sources, like spot size and shape variation and bleeding are included in simulation. On the slide image on the right (b) also unideal subarray alignment and scratches are introduced.

**Figure 9:**
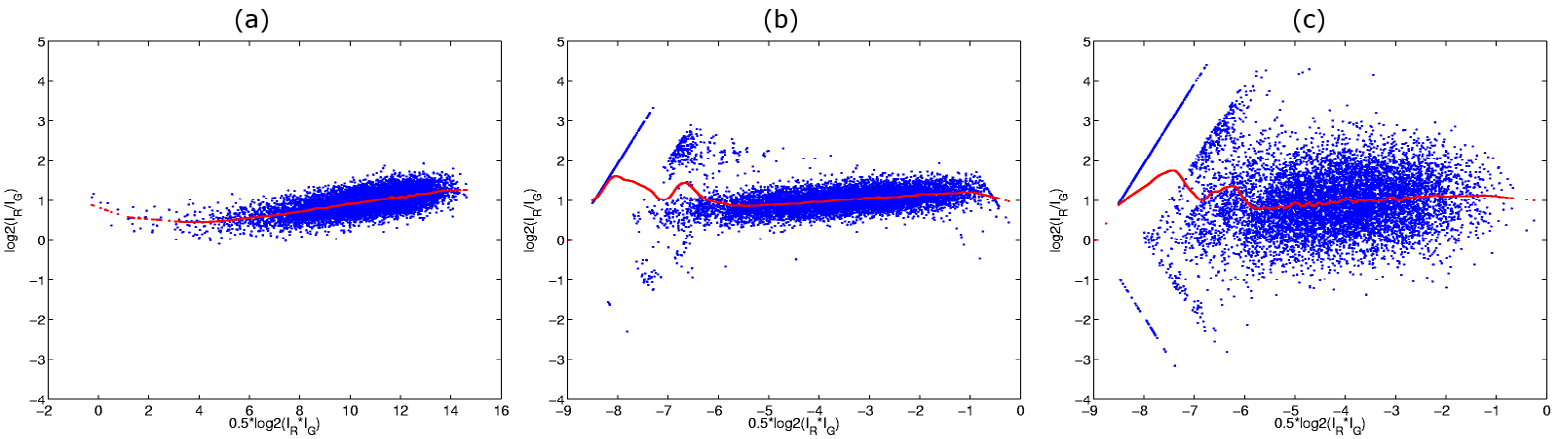
**Scatter plots of the simulated data**. MA-plot is shown for (a) noise free simulated data, (b) noise free data extracted from slide, and (c) realistic simulated data. Lowess fit is shown over each scatter plot to illustrate the trends in the data. All data points extracted from slide are scaled to [0,1] interval, thus the scale of the x-axis is different to the simulated noise free data.

After the error model is applied, we generate the slide images. As an example we show two slide images in Figure 8, generated at time instants 10 and 200 minutes corresponding to the time scale in Figures 6 and 7. It can be observed that in the beginning only one spot shows a difference in the expression. This corresponds to the gene that was knocked out. At the later time instant the effect of the knockout has spread through the network, and many genes show change in their expression. That is, spot colors have changed from yellow to red or green. In this example the amount of biological and measurement errors is in the minimum in order to point out the spreading effects of the knock out. Adding more measurement and biological errors would introduce more changes in the expressions already at the 10 minutes time instant. To illustrate that the simulated data has properties similar to real microarray data, we show scatter plots of the simulated data. For this example the ground truth data is drawn from predetermined distribution. Common assumption is that the ground truth expression values are from an exponential distribution *I *= *λe*^-*λx *^[7,15]. We draw 10000 expression values from this distribution, with *λ *= 1/3000. As we are interested in evaluating the quality of the data, we do not introduce any differentially expressed genes, but simulate a self versus self experiment as explained in [15]. Red and green intensities *I*_*R *_and *I*_*G *_are drawn from a normal distribution *N *(*I*, *αI*), where *α *= 0.1 and *I *is a realization from an exponential distribution. Next, the final red and green intensities *I*_*R *_and *I*_*G *_are transformed with x^=xa0+a1
 MathType@MTEF@5@5@+=feaafiart1ev1aaatCvAUfKttLearuWrP9MDH5MBPbIqV92AaeXatLxBI9gBaebbnrfifHhDYfgasaacH8akY=wiFfYdH8Gipec8Eeeu0xXdbba9frFj0=OqFfea0dXdd9vqai=hGuQ8kuc9pgc9s8qqaq=dirpe0xb9q8qiLsFr0=vr0=vr0dc8meaabaqaciaacaGaaeqabaqabeGadaaakeaacuWG4baEgaqcaiabg2da9iabdIha4naaCaaaleqabaGaemyyae2aaSbaaWqaaiabicdaWaqabaaaaOGaey4kaSIaemyyae2aaSbaaSqaaiabigdaXaqabaaaaa@369A@, where *x *= {*I*_*R*_, *I*_*G*_} with parameters *a*_0 _= 1.04, *a*_1 _= 0.5 for *I*_*R *_and *a*_0 _= 0.95, *a*_1 _= -0.2 for *I*_*G*_. This is a simplified version of the ground truth data generation proposed in [15]. Scatter plot of the data is shown in Figure 9(a). To illustrate what is the effect of reading the spot values from a simulated slide image, we simulate a microarray without adding any biological or measurement errors to the ground truth data. Only slide manufacturing and hybridization errors are introduced. Scatter plot of the noise free data extracted from a simulated slide image is shown in Figure 9(b). It can be observed that the extraction of the data alone introduces some errors.

**Figure 10:**
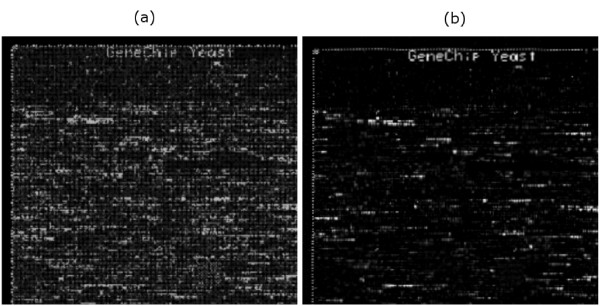
**Example of an Affymetrix microarray simulation**. Example of the simulated single-channel oligonucleotide microarray slide image (crop from top left corner) (a). We have used an Affymetrix .cel file as the ground truth data. Thus the text about the slide type is observable. Real Affymetrix slide image is shown for comparison (b).

Finally, we run the simulated ground truth data through a hierarchical error model [6]. The resulting scatter plot is shown in Figure 9(c). It is difficult to quantify objectively if the data is really realistic, but the scatter plot shows characteristics that are observed from real microarray data [2,32]. For example, the arrow head shape at the left is observed with real microarray data [32]. Furthermore, the scatter pattern shows more variation at the small intensity values, which is the case with real microarray data also [32]. As another example we will demonstrate the simulation of a single-channel oligonucleotide microarray slide. As the ground truth data we use yeast data that can be downloaded from Affymetrix web site [33]. We simulate a slide image based on the intensity values in the .cel file. Figure 10 represents a crop of the simulated image and the corresponding real image from the original .dat file.

As the final application example, we present how the proposed simulation model can be used for comparing spot segmentation algorithms. Spot segmentation, along with procedures such as spot addressing and estimation of background and foreground levels, is one of the successive steps affecting the estimation of the true signal intensity. Simultaneous comparison of all the methods affecting the estimated true signal is a complex problem which would require more attention in order to be thoroughly studied. In our current example we estimate the spot and the background intensities by calculating the mean of segmented foreground and background pixels. Thereafter, the expression value is obtained by subtracting the background intensities from the foreground intensities. Our comparison example includes three different segmentation algorithms: The fixed circle (FC) method [34], the histogram segmentation (HST) method [35], and the seeded region growing (SRG) method [36].

**Figure 11:**
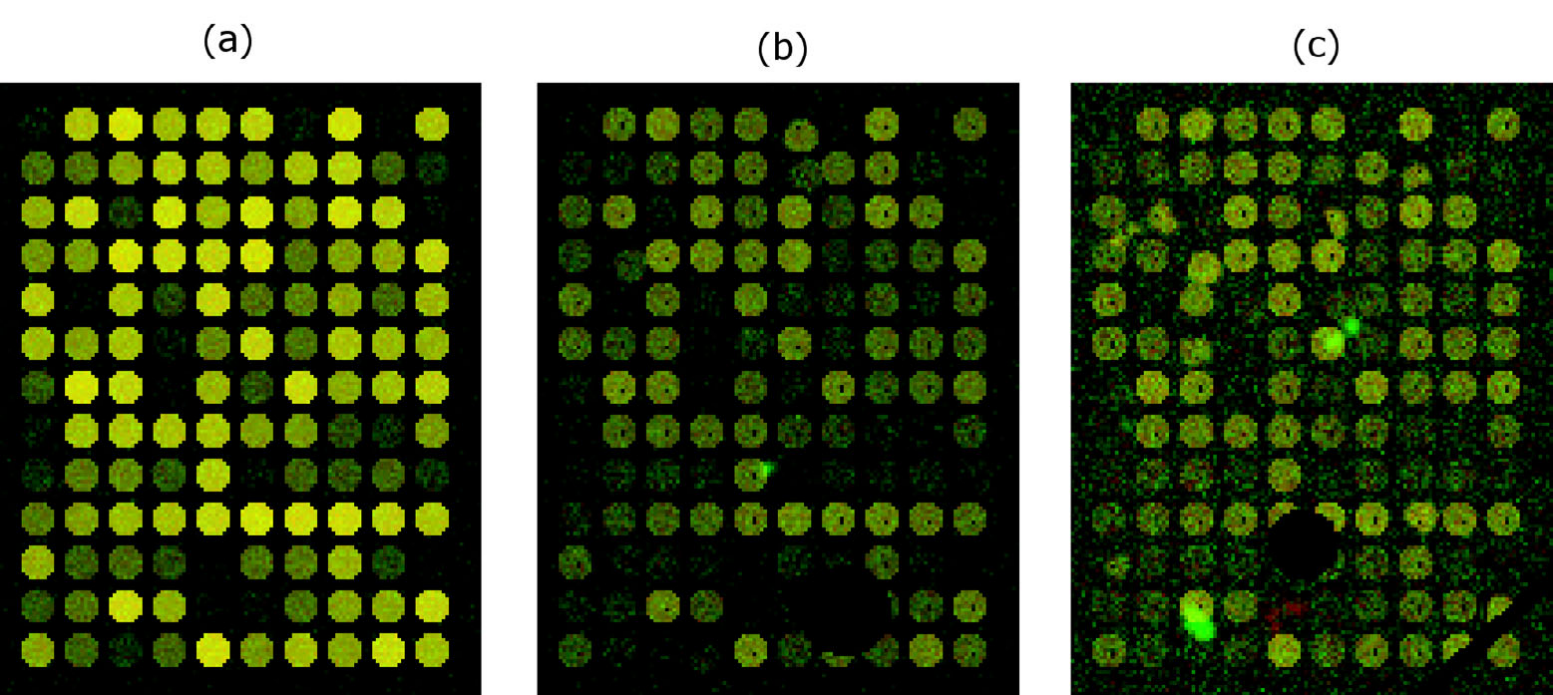
**Slide image segmentation examples**. One subarray from each of the images used to test the segmentation algorithms are shown. From left to right: (a) high quality slide, (b) noisy slide with artifacts, and (c) disturbing noise and artifacts over the slide. Increase in noise and degradation of the spot quality is clearly observable.

We simulate three test images consisting of eight subarrays with altogether 1000 spots per image. Each image has different quality characteristics. The first image is of high quality, with low variance noise (0.01) and relatively round and regularly sized spots. The second image has more noise (variance 0.02) and more irregular spot shapes and sizes, while the third has even more disturbing noise which has higher variance (0.03). Furthermore the spot shapes and sizes include more variation compared to the other images. Air bubbles, scratches, spot bleeding, and print tip effects are added into the second and third image, the third including more such artefacts than the second image. Figure 11 shows one subarray from each of the images used in this experiment. Detailed information about the simulation parameters for these three images is available on the companion web page.

**Figure 12:**
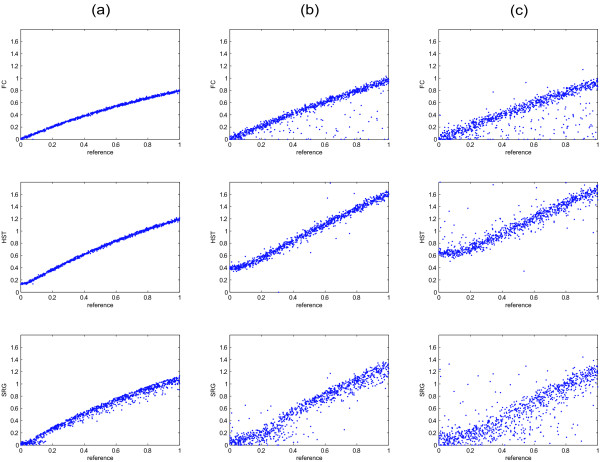
**Results of segmentation example**. The spot intensities estimated from the simulated images with the fixed circle (first row), the histogram segmentation (second row), and the seeded region growing (third row) segmentation algorithms are plotted against the input data (reference). The plots are from the first channel of the test images: (a) intensities for the high quality image given by the three segmentation algorithms, (b) intensity plots for image with noise and errors, (c) plots for image with disturbing noise and artefacts.

The results of applying the selected segmentation algorithms on the synthetic test images and calculating the spot intensities from the segmentation results are shown in Figure 12 where the estimated spot intensities are plotted against the reference signal. Figure 12(a) shows the scatter plots for the first image, 12(b) shows the plots for the second image of slightly degraded quality, and 12(c) presents the plots for the third, low quality image. After removing the estimated background, some of the spot intensities become negative. These negative intensities are replaced with zeros. To quantify the performance of different algorithms, we compute the correlation coefficient for each comparison. The results are given in Table 7. Even though we mainly focus on simulating images with realistic parameters, some observations on the segmentation results are presented. The results presented in Table 7 support intuition; all methods give worse results as the image quality is degraded. The fixed circle segmentation is likely to be confused by the irregular shapes and sizes of the spots in the second image (shown in Figure 11(b)) and especially in the third image (shown in Figure 11(c)). The other methods are corrupted mainly by the noise in the second and the third image. Despite the high correlation with the reference expressions, the intensity given by HST segmentation method suffers from a relatively high bias. However, the low scattering of intensities given by HST, compared to that of FC and SRG, explains the high correlation. HST has also less outliers on the lower side of the scatter plot. Both the bias in HST and scattering in FC and SRG are clearly visible in Figure 12. The results of the segmentation experiment are well in accordance with the basic assumptions. Thus, the images produced by the proposed simulation model can be used for testing microarray image processing algorithms, and the model provides useful information about the available methods.

## Conclusion

The previously proposed microarray simulation models have been suitable for specific simulation tasks only. The model we have proposed is modular and can be used in different kinds of analyzes. One of the most important properties of the proposed model is the ability to use almost any kind of input data. Most models are limited to specific types of data, typically random data drawn from a predetermined distribution. Thus, they can not exploit other data, such as data produced by network simulation. In addition, the proposed model utilizes several previously published error models in modeling the biological and measurement technology dependent variation. Thus, the model is not dependent of any specific formulation of noise characteristics, and the performance of the analysis algorithms can effectively be tested under different noise assumptions. Our model also supports both spotted two-channel and oligonucleotide based single-channel microarrays.

We have shown that the proposed model can be used to simulate microarray data which is valuable for validating various kind of data analysis algorithms. As an example, the performance of the microarray segmentation algorithms were compared under different noise conditions.

## Authors' contributions

MN and MA designed and implemented the microarray simulation model. TA was responsible for simulating the biological ground truth data. PR and AL performed image processing experiments. OY-H conceived of the study and participated in the design and coordination. MN drafted the manuscript. All authors read and approved the final manuscript.

## Supplementary Material

Additional file 1 Microarray simulation model. Matlab implementation of the microarray simulation model. Click here for file

## Figures and Tables

**Table 1:** List of noise parameters. Noise parameters available in the microarray simulation model.

kernel	Kernel used to model the population effect.
copies	Number of times the population effect is applied.
errormodel	Error model to be used; each error model has its own parameters, see Table 5.

**Table 2:** List of slide parameters. Overview of the slide simulation parameters. More detailed documentation of the parameters is available on the companion web page [19].

*S*_*type*_	Type of the slide (single or two channel).
*S*_*spot*_	Model used for the spot: circle, Gaussian, hyperbolic.
*S*_*pix*_	Maximum width/height of the area for the spot in pixels.
*S*_*movprob*_	Probability for a spot to drift (move) from designated location. This parameter models random movement. See parameter *B*_*curve *_for systematic drift.
*S*_*mov*_	Maximum allowed movement bias from designated location, movement in x-axis *S*_*x *_and y-axis *S*_*y *_are drawn from uniform distribution *U *(-*S*_*mov*_, *S*_*mov*_).
*S*_*μ*_	Mean radius of the simulated spot. Spot radius is drawn from *N *(*S*_*μ*_, Sσ2 MathType@MTEF@5@5@+=feaafiart1ev1aaatCvAUfKttLearuWrP9MDH5MBPbIqV92AaeXatLxBI9gBaebbnrfifHhDYfgasaacH8akY=wiFfYdH8Gipec8Eeeu0xXdbba9frFj0=OqFfea0dXdd9vqai=hGuQ8kuc9pgc9s8qqaq=dirpe0xb9q8qiLsFr0=vr0=vr0dc8meaabaqaciaacaGaaeqabaqabeGadaaakeaacqWGtbWudaWgaaWcbaacciGae83Wdm3aaWbaaWqabeaacqaIYaGmaaaaleqaaaaa@30FC@) distribution.
Sσ2 MathType@MTEF@5@5@+=feaafiart1ev1aaatCvAUfKttLearuWrP9MDH5MBPbIqV92AaeXatLxBI9gBaebbnrfifHhDYfgasaacH8akY=wiFfYdH8Gipec8Eeeu0xXdbba9frFj0=OqFfea0dXdd9vqai=hGuQ8kuc9pgc9s8qqaq=dirpe0xb9q8qiLsFr0=vr0=vr0dc8meaabaqaciaacaGaaeqabaqabeGadaaakeaacqWGtbWudaWgaaWcbaacciGae83Wdm3aaWbaaWqabeaacqaIYaGmaaaaleqaaaaa@30FC@	Allowed variation (variance) of the spot size.
*P*	If set, print tip leaves a mark to the spot.
*P*_*p*_	Probability for print tip mark to be visible in a spot.
*P*_*h*_	Maximum height of the print tip mark, print tip height is drawn from *U *(0, *P*_*h*_) distribution.
*P*_*w*_	Maximum width of the print tip mark, print tip width is drawn from *U *(0, *P*_*w*_) distribution.
*P*_*b*_	Maximum of how much print tip mark is allowed to drift from spot center. Movement in x-axis *P*_*x *_and y-axis *P*_*y *_are drawn from *U *(-*P*_*b*_, *P*_*b*_).
*C*_*prob*_	Probability for a spot to suffer from a chord cut.
*C*_*num*_	Maximum number of chord cuts from a spot.
*C*_*cut*_	Maximum depth of the chord cut, cut depth is drawn from *U *(0, *C*_*cut*_).
*N*_*slides*_	Number of slides to be generated.
*N*_*time*_	Time points when slides are made. This is relevant only for time series data.
*N*_*channels*_	Number of channels (different dyes) on the slide.
*N*_*spots*_	Total number of spots on the slide.
*N*_*height*_	Number of rows of spots on the slide.
*N*_*width*_	Number of columns of spots on the slide.
*B*	Subarray layout on the slide i.e. number of (subarray)rows and (subarray)columns.
*B*_*space*_	Space between individual subarrays on the slide.
*B*_*curve*_	Parameter used to control the subarray curving (i.e. systematic drift in spot printing).
*B*_*maxc*_	Maximum distance the bin is allowed to curve, curvature parameter is drawn from *U *(0, *B*_*maxc*_).
*B*_*spots*_	Number of spots in each subarray.
*B*_*height*_	Number of rows in subarrays.
*B*_*width*_	Number of columns in subarrays.

**Table 3:** List of hybridization parameters. Overview of the hybridization effect parameters.

Hσ2 MathType@MTEF@5@5@+=feaafiart1ev1aaatCvAUfKttLearuWrP9MDH5MBPbIqV92AaeXatLxBI9gBaebbnrfifHhDYfgasaacH8akY=wiFfYdH8Gipec8Eeeu0xXdbba9frFj0=OqFfea0dXdd9vqai=hGuQ8kuc9pgc9s8qqaq=dirpe0xb9q8qiLsFr0=vr0=vr0dc8meaabaqaciaacaGaaeqabaqabeGadaaakeaacqWGibasdaWgaaWcbaacciGae83Wdm3aaWbaaWqabeaacqaIYaGmaaaaleqaaaaa@30E6@	Multiplicative Gaussian hybridization noise variance. Hybridization noise is drawn from *N *(0, Hσ2 MathType@MTEF@5@5@+=feaafiart1ev1aaatCvAUfKttLearuWrP9MDH5MBPbIqV92AaeXatLxBI9gBaebbnrfifHhDYfgasaacH8akY=wiFfYdH8Gipec8Eeeu0xXdbba9frFj0=OqFfea0dXdd9vqai=hGuQ8kuc9pgc9s8qqaq=dirpe0xb9q8qiLsFr0=vr0=vr0dc8meaabaqaciaacaGaaeqabaqabeGadaaakeaacqWGibasdaWgaaWcbaacciGae83Wdm3aaWbaaWqabeaacqaIYaGmaaaaleqaaaaa@30E6@).
*H*_*errors*_	If set, hybridization errors are included in simulation.
*H*_*bgnoise*_	Percent of the intensity values covered by the background noise.
*H*_*bgvar*_	Background noise variance, relative to background noise mean determined using *H*_*bgnoise*_.
*H*_*bggrad*_	Gradient (noise pattern) for background noise.
*H*_*noscratch*_	Number of scratches on the slide.
*H*_*slength*_	Maximum length of the scratch, scratch length is drawn from *U *(0, *H*_*Slength*_).
*H*_*Swidth*_	Width of the scratch.
*H*_*noair*_	Number of air bubbles visible on the slide.
Hμair MathType@MTEF@5@5@+=feaafiart1ev1aaatCvAUfKttLearuWrP9MDH5MBPbIqV92AaeXatLxBI9gBaebbnrfifHhDYfgasaacH8akY=wiFfYdH8Gipec8Eeeu0xXdbba9frFj0=OqFfea0dXdd9vqai=hGuQ8kuc9pgc9s8qqaq=dirpe0xb9q8qiLsFr0=vr0=vr0dc8meaabaqaciaacaGaaeqabaqabeGadaaakeaacqWGibasdaWgaaWcbaacciGae8hVd02aaSbaaWqaaiabdggaHjabdMgaPjabdkhaYbqabaaaleqaaaaa@33F9@	Mean for the air bubble radius, drawn from *N *(*μ*_*air*_, σair2 MathType@MTEF@5@5@+=feaafiart1ev1aaatCvAUfKttLearuWrP9MDH5MBPbIqV92AaeXatLxBI9gBaebbnrfifHhDYfgasaacH8akY=wiFfYdH8Gipec8Eeeu0xXdbba9frFj0=OqFfea0dXdd9vqai=hGuQ8kuc9pgc9s8qqaq=dirpe0xb9q8qiLsFr0=vr0=vr0dc8meaabaqaciaacaGaaeqabaqabeGadaaakeaaiiGacqWFdpWCdaqhaaWcbaGaemyyaeMaemyAaKMaemOCaihabaGaeGOmaidaaaaa@33A8@).
Hσair2 MathType@MTEF@5@5@+=feaafiart1ev1aaatCvAUfKttLearuWrP9MDH5MBPbIqV92AaeXatLxBI9gBaebbnrfifHhDYfgasaacH8akY=wiFfYdH8Gipec8Eeeu0xXdbba9frFj0=OqFfea0dXdd9vqai=hGuQ8kuc9pgc9s8qqaq=dirpe0xb9q8qiLsFr0=vr0=vr0dc8meaabaqaciaacaGaaeqabaqabeGadaaakeaacqWGibasdaWgaaWcbaacciGae83Wdm3aa0baaWqaaiabdggaHjabdMgaPjabdkhaYbqaaiabikdaYaaaaSqabaaaaa@34F9@	Allowed variation (variance) for air bubble size radius.
*H*_*bleed*_	Percent of spots having dye outside spot area (bleeding).
*H*_*bleedsize*_	Size of the spot bleed (how many times the spot size).
*H*_*bleeddist*_	How far from the origin the bleeding goes.

**Table 4:** List of scanner parameters. Overview of the scanner effect parameters.

*R*_*power*_	Scanner power is used for histogram equalization, more power yields brighter image.
*R*_*b*_	The dynamic range of the scanner. Intensity values are quantized to 2Rb MathType@MTEF@5@5@+=feaafiart1ev1aaatCvAUfKttLearuWrP9MDH5MBPbIqV92AaeXatLxBI9gBaebbnrfifHhDYfgasaacH8akY=wiFfYdH8Gipec8Eeeu0xXdbba9frFj0=OqFfea0dXdd9vqai=hGuQ8kuc9pgc9s8qqaq=dirpe0xb9q8qiLsFr0=vr0=vr0dc8meaabaqaciaacaGaaeqabaqabeGadaaakeaacqaIYaGmdaahaaWcbeqaaiabdkfasnaaBaaameaacqWGIbGyaeqaaaaaaaa@3072@ interval.
*R*_*eq*_	If set, histogram equalization is applied.
*R*_*th*_	Threshold parameter for quantization, values over the threshold are saturated.
*R*_*Rch*_	Number of channel that is considered as red dye.
*R*_*Gch*_	Number of channel that is considered as green dye.
*R*_*errors*_	If set, scanner errors are applied.
*R*_*angle*_	Angle at which the slide is scanned.
*R*_*mm*_	Misalignment between red and green channel.

**Table 5:** List of error models. Error models (EM) and the parameters for each of the implemented error model. Noise free input data is denoted by *x *and the noisy output data by *y*. Index *i *refers to gene, *j *to array (chip), and *k *to biological sample specific noise. Index *p *refers to a specific probe within a probe set.

**Simple EM**:	
Model	Additive Gaussian noise is added to the data.
*μ*	Mean of the additive Gaussian noise. Noise is drawn from *N *(*μ*, *α*^2^)
*α*^2^	Variance of the additive Gaussian noise.
**SNR EM**:	
Model	Additive Gaussian noise is added to the data with given signal-to-noise ratio.
*μ*	Mean of the additive Gaussian noise.
SNR	Signal-to-noise ratio after the noise is added.
**Dror EM **[7]:	
Model	*y *= g * (*x*_i _* *x*) + *f *+ *ε*
μxi MathType@MTEF@5@5@+=feaafiart1ev1aaatCvAUfKttLearuWrP9MDH5MBPbIqV92AaeXatLxBI9gBaebbnrfifHhDYfgasaacH8akY=wiFfYdH8Gipec8Eeeu0xXdbba9frFj0=OqFfea0dXdd9vqai=hGuQ8kuc9pgc9s8qqaq=dirpe0xb9q8qiLsFr0=vr0=vr0dc8meaabaqaciaacaGaaeqabaqabeGadaaakeaaiiGacqWF8oqBdaWgaaWcbaGaemiEaG3aaSbaaWqaaiabdMgaPbqabaaaleqaaaaa@31A1@, σxi2 MathType@MTEF@5@5@+=feaafiart1ev1aaatCvAUfKttLearuWrP9MDH5MBPbIqV92AaeXatLxBI9gBaebbnrfifHhDYfgasaacH8akY=wiFfYdH8Gipec8Eeeu0xXdbba9frFj0=OqFfea0dXdd9vqai=hGuQ8kuc9pgc9s8qqaq=dirpe0xb9q8qiLsFr0=vr0=vr0dc8meaabaqaciaacaGaaeqabaqabeGadaaakeaaiiGacqWFdpWCdaqhaaWcbaGaemiEaG3aaSbaaWqaaiabdMgaPbqabaaaleaacqaIYaGmaaaaaa@32A1@	Binding efficiency of each probe *x*_*i *_is drawn from Gaussian distribution *N *(μxi MathType@MTEF@5@5@+=feaafiart1ev1aaatCvAUfKttLearuWrP9MDH5MBPbIqV92AaeXatLxBI9gBaebbnrfifHhDYfgasaacH8akY=wiFfYdH8Gipec8Eeeu0xXdbba9frFj0=OqFfea0dXdd9vqai=hGuQ8kuc9pgc9s8qqaq=dirpe0xb9q8qiLsFr0=vr0=vr0dc8meaabaqaciaacaGaaeqabaqabeGadaaakeaaiiGacqWF8oqBdaWgaaWcbaGaemiEaG3aaSbaaWqaaiabdMgaPbqabaaaleqaaaaa@31A1@, σxi2 MathType@MTEF@5@5@+=feaafiart1ev1aaatCvAUfKttLearuWrP9MDH5MBPbIqV92AaeXatLxBI9gBaebbnrfifHhDYfgasaacH8akY=wiFfYdH8Gipec8Eeeu0xXdbba9frFj0=OqFfea0dXdd9vqai=hGuQ8kuc9pgc9s8qqaq=dirpe0xb9q8qiLsFr0=vr0=vr0dc8meaabaqaciaacaGaaeqabaqabeGadaaakeaaiiGacqWFdpWCdaqhaaWcbaGaemiEaG3aaSbaaWqaaiabdMgaPbqabaaaleaacqaIYaGmaaaaaa@32A1@).
*μ*_*f*_, σf2 MathType@MTEF@5@5@+=feaafiart1ev1aaatCvAUfKttLearuWrP9MDH5MBPbIqV92AaeXatLxBI9gBaebbnrfifHhDYfgasaacH8akY=wiFfYdH8Gipec8Eeeu0xXdbba9frFj0=OqFfea0dXdd9vqai=hGuQ8kuc9pgc9s8qqaq=dirpe0xb9q8qiLsFr0=vr0=vr0dc8meaabaqaciaacaGaaeqabaqabeGadaaakeaaiiGacqWFdpWCdaqhaaWcbaGaemOzaygabaGaeGOmaidaaaaa@30EA@	Gene specific bias *f *is drawn from Gaussian distribution *N *(*μ*_*f*_, σf2 MathType@MTEF@5@5@+=feaafiart1ev1aaatCvAUfKttLearuWrP9MDH5MBPbIqV92AaeXatLxBI9gBaebbnrfifHhDYfgasaacH8akY=wiFfYdH8Gipec8Eeeu0xXdbba9frFj0=OqFfea0dXdd9vqai=hGuQ8kuc9pgc9s8qqaq=dirpe0xb9q8qiLsFr0=vr0=vr0dc8meaabaqaciaacaGaaeqabaqabeGadaaakeaaiiGacqWFdpWCdaqhaaWcbaGaemOzaygabaGaeGOmaidaaaaa@30EA@).
*α*_*ε*_, *β*_*ε*_	Gene and chip specific error *ε *is drawn from Laplace distribution *L *(*α*_*ε*_, *β*_*ε*_).
*μ*_*g*_, σg2 MathType@MTEF@5@5@+=feaafiart1ev1aaatCvAUfKttLearuWrP9MDH5MBPbIqV92AaeXatLxBI9gBaebbnrfifHhDYfgasaacH8akY=wiFfYdH8Gipec8Eeeu0xXdbba9frFj0=OqFfea0dXdd9vqai=hGuQ8kuc9pgc9s8qqaq=dirpe0xb9q8qiLsFr0=vr0=vr0dc8meaabaqaciaacaGaaeqabaqabeGadaaakeaaiiGacqWFdpWCdaqhaaWcbaGaem4zaCgabaGaeGOmaidaaaaa@30EC@	Multiplicative gene and chip specific noise *g *is drawn from log-normal distribution *LN *(*μ*_*g*_, σg2 MathType@MTEF@5@5@+=feaafiart1ev1aaatCvAUfKttLearuWrP9MDH5MBPbIqV92AaeXatLxBI9gBaebbnrfifHhDYfgasaacH8akY=wiFfYdH8Gipec8Eeeu0xXdbba9frFj0=OqFfea0dXdd9vqai=hGuQ8kuc9pgc9s8qqaq=dirpe0xb9q8qiLsFr0=vr0=vr0dc8meaabaqaciaacaGaaeqabaqabeGadaaakeaaiiGacqWFdpWCdaqhaaWcbaGaem4zaCgabaGaeGOmaidaaaaa@30EC@).
**Hartemink EM **[9]:	
Model	In log scale *y *= *x *+ *ρ*_*j *_+ *ε*_*ij*_
μpj MathType@MTEF@5@5@+=feaafiart1ev1aaatCvAUfKttLearuWrP9MDH5MBPbIqV92AaeXatLxBI9gBaebbnrfifHhDYfgasaacH8akY=wiFfYdH8Gipec8Eeeu0xXdbba9frFj0=OqFfea0dXdd9vqai=hGuQ8kuc9pgc9s8qqaq=dirpe0xb9q8qiLsFr0=vr0=vr0dc8meaabaqaciaacaGaaeqabaqabeGadaaakeaaiiGacqWF8oqBdaWgaaWcbaGaemiCaa3aaSbaaWqaaiabdQgaQbqabaaaleqaaaaa@3193@, σpj2 MathType@MTEF@5@5@+=feaafiart1ev1aaatCvAUfKttLearuWrP9MDH5MBPbIqV92AaeXatLxBI9gBaebbnrfifHhDYfgasaacH8akY=wiFfYdH8Gipec8Eeeu0xXdbba9frFj0=OqFfea0dXdd9vqai=hGuQ8kuc9pgc9s8qqaq=dirpe0xb9q8qiLsFr0=vr0=vr0dc8meaabaqaciaacaGaaeqabaqabeGadaaakeaaiiGacqWFdpWCdaqhaaWcbaGaemiCaa3aaSbaaWqaaiabdQgaQbqabaaaleaacqaIYaGmaaaaaa@3293@	Chip specific bias *ρ*_*j *_is drawn from Gaussian distribution *N *(μpj MathType@MTEF@5@5@+=feaafiart1ev1aaatCvAUfKttLearuWrP9MDH5MBPbIqV92AaeXatLxBI9gBaebbnrfifHhDYfgasaacH8akY=wiFfYdH8Gipec8Eeeu0xXdbba9frFj0=OqFfea0dXdd9vqai=hGuQ8kuc9pgc9s8qqaq=dirpe0xb9q8qiLsFr0=vr0=vr0dc8meaabaqaciaacaGaaeqabaqabeGadaaakeaaiiGacqWF8oqBdaWgaaWcbaGaemiCaa3aaSbaaWqaaiabdQgaQbqabaaaleqaaaaa@3193@, σpj2 MathType@MTEF@5@5@+=feaafiart1ev1aaatCvAUfKttLearuWrP9MDH5MBPbIqV92AaeXatLxBI9gBaebbnrfifHhDYfgasaacH8akY=wiFfYdH8Gipec8Eeeu0xXdbba9frFj0=OqFfea0dXdd9vqai=hGuQ8kuc9pgc9s8qqaq=dirpe0xb9q8qiLsFr0=vr0=vr0dc8meaabaqaciaacaGaaeqabaqabeGadaaakeaaiiGacqWFdpWCdaqhaaWcbaGaemiCaa3aaSbaaWqaaiabdQgaQbqabaaaleaacqaIYaGmaaaaaa@3293@).
σεij2 MathType@MTEF@5@5@+=feaafiart1ev1aaatCvAUfKttLearuWrP9MDH5MBPbIqV92AaeXatLxBI9gBaebbnrfifHhDYfgasaacH8akY=wiFfYdH8Gipec8Eeeu0xXdbba9frFj0=OqFfea0dXdd9vqai=hGuQ8kuc9pgc9s8qqaq=dirpe0xb9q8qiLsFr0=vr0=vr0dc8meaabaqaciaacaGaaeqabaqabeGadaaakeaaiiGacqWFdpWCdaqhaaWcbaGae8xTdu2aaSbaaWqaaiabdMgaPjabdQgaQbqabaaaleaacqaIYaGmaaaaaa@3427@	Gene and chip specific error *ε*_*ij *_is drawn from Gaussian distribution *N *(0, σeij2 MathType@MTEF@5@5@+=feaafiart1ev1aaatCvAUfKttLearuWrP9MDH5MBPbIqV92AaeXatLxBI9gBaebbnrfifHhDYfgasaacH8akY=wiFfYdH8Gipec8Eeeu0xXdbba9frFj0=OqFfea0dXdd9vqai=hGuQ8kuc9pgc9s8qqaq=dirpe0xb9q8qiLsFr0=vr0=vr0dc8meaabaqaciaacaGaaeqabaqabeGadaaakeaaiiGacqWFdpWCdaqhaaWcbaGaemyzau2aaSbaaWqaaiabdMgaPjabdQgaQbqabaaaleaacqaIYaGmaaaaaa@33D8@).
**Hierarchical EM **[6]:	
Model	In log scale *y *= *X *+ *ε*, *X *= *x *+ *g*_*i *_+ *c*_*j*_, + *r*_*ij *_+ *b*_*ijk*_
σε2 MathType@MTEF@5@5@+=feaafiart1ev1aaatCvAUfKttLearuWrP9MDH5MBPbIqV92AaeXatLxBI9gBaebbnrfifHhDYfgasaacH8akY=wiFfYdH8Gipec8Eeeu0xXdbba9frFj0=OqFfea0dXdd9vqai=hGuQ8kuc9pgc9s8qqaq=dirpe0xb9q8qiLsFr0=vr0=vr0dc8meaabaqaciaacaGaaeqabaqabeGadaaakeaaiiGacqWFdpWCdaqhaaWcbaGae8xTdugabaGaeGOmaidaaaaa@3137@	Independent random noise *ε *is drawn from zero mean Gaussian distribution *N *(0, σε2 MathType@MTEF@5@5@+=feaafiart1ev1aaatCvAUfKttLearuWrP9MDH5MBPbIqV92AaeXatLxBI9gBaebbnrfifHhDYfgasaacH8akY=wiFfYdH8Gipec8Eeeu0xXdbba9frFj0=OqFfea0dXdd9vqai=hGuQ8kuc9pgc9s8qqaq=dirpe0xb9q8qiLsFr0=vr0=vr0dc8meaabaqaciaacaGaaeqabaqabeGadaaakeaaiiGacqWFdpWCdaqhaaWcbaGae8xTdugabaGaeGOmaidaaaaa@3137@).
σgi2 MathType@MTEF@5@5@+=feaafiart1ev1aaatCvAUfKttLearuWrP9MDH5MBPbIqV92AaeXatLxBI9gBaebbnrfifHhDYfgasaacH8akY=wiFfYdH8Gipec8Eeeu0xXdbba9frFj0=OqFfea0dXdd9vqai=hGuQ8kuc9pgc9s8qqaq=dirpe0xb9q8qiLsFr0=vr0=vr0dc8meaabaqaciaacaGaaeqabaqabeGadaaakeaaiiGacqWFdpWCdaqhaaWcbaGaem4zaC2aaSbaaWqaaiabdMgaPbqabaaaleaacqaIYaGmaaaaaa@327F@	Gene specific noise *g*_*i *_is drawn from zero mean Gaussian distribution.*N *(0, σgi2 MathType@MTEF@5@5@+=feaafiart1ev1aaatCvAUfKttLearuWrP9MDH5MBPbIqV92AaeXatLxBI9gBaebbnrfifHhDYfgasaacH8akY=wiFfYdH8Gipec8Eeeu0xXdbba9frFj0=OqFfea0dXdd9vqai=hGuQ8kuc9pgc9s8qqaq=dirpe0xb9q8qiLsFr0=vr0=vr0dc8meaabaqaciaacaGaaeqabaqabeGadaaakeaaiiGacqWFdpWCdaqhaaWcbaGaem4zaC2aaSbaaWqaaiabdMgaPbqabaaaleaacqaIYaGmaaaaaa@327F@).
σcj2 MathType@MTEF@5@5@+=feaafiart1ev1aaatCvAUfKttLearuWrP9MDH5MBPbIqV92AaeXatLxBI9gBaebbnrfifHhDYfgasaacH8akY=wiFfYdH8Gipec8Eeeu0xXdbba9frFj0=OqFfea0dXdd9vqai=hGuQ8kuc9pgc9s8qqaq=dirpe0xb9q8qiLsFr0=vr0=vr0dc8meaabaqaciaacaGaaeqabaqabeGadaaakeaaiiGacqWFdpWCdaqhaaWcbaGaem4yam2aaSbaaWqaaiabdQgaQbqabaaaleaacqaIYaGmaaaaaa@3279@	Chip specific noise *C*_*j *_is drawn from zero mean Gaussian distribution *N *(0, σcj2 MathType@MTEF@5@5@+=feaafiart1ev1aaatCvAUfKttLearuWrP9MDH5MBPbIqV92AaeXatLxBI9gBaebbnrfifHhDYfgasaacH8akY=wiFfYdH8Gipec8Eeeu0xXdbba9frFj0=OqFfea0dXdd9vqai=hGuQ8kuc9pgc9s8qqaq=dirpe0xb9q8qiLsFr0=vr0=vr0dc8meaabaqaciaacaGaaeqabaqabeGadaaakeaaiiGacqWFdpWCdaqhaaWcbaGaem4yam2aaSbaaWqaaiabdQgaQbqabaaaleaacqaIYaGmaaaaaa@3279@).
σrij2 MathType@MTEF@5@5@+=feaafiart1ev1aaatCvAUfKttLearuWrP9MDH5MBPbIqV92AaeXatLxBI9gBaebbnrfifHhDYfgasaacH8akY=wiFfYdH8Gipec8Eeeu0xXdbba9frFj0=OqFfea0dXdd9vqai=hGuQ8kuc9pgc9s8qqaq=dirpe0xb9q8qiLsFr0=vr0=vr0dc8meaabaqaciaacaGaaeqabaqabeGadaaakeaaiiGacqWFdpWCdaqhaaWcbaGaemOCai3aaSbaaWqaaiabdMgaPjabdQgaQbqabaaaleaacqaIYaGmaaaaaa@33F2@	Gene and chip specific noise *r*_*ij *_is drawn from zero mean Gaussian distribution *N *(0, σrij2 MathType@MTEF@5@5@+=feaafiart1ev1aaatCvAUfKttLearuWrP9MDH5MBPbIqV92AaeXatLxBI9gBaebbnrfifHhDYfgasaacH8akY=wiFfYdH8Gipec8Eeeu0xXdbba9frFj0=OqFfea0dXdd9vqai=hGuQ8kuc9pgc9s8qqaq=dirpe0xb9q8qiLsFr0=vr0=vr0dc8meaabaqaciaacaGaaeqabaqabeGadaaakeaaiiGacqWFdpWCdaqhaaWcbaGaemOCai3aaSbaaWqaaiabdMgaPjabdQgaQbqabaaaleaacqaIYaGmaaaaaa@33F2@).
σbijk2 MathType@MTEF@5@5@+=feaafiart1ev1aaatCvAUfKttLearuWrP9MDH5MBPbIqV92AaeXatLxBI9gBaebbnrfifHhDYfgasaacH8akY=wiFfYdH8Gipec8Eeeu0xXdbba9frFj0=OqFfea0dXdd9vqai=hGuQ8kuc9pgc9s8qqaq=dirpe0xb9q8qiLsFr0=vr0=vr0dc8meaabaqaciaacaGaaeqabaqabeGadaaakeaaiiGacqWFdpWCdaqhaaWcbaGaemOyai2aaSbaaWqaaiabdMgaPjabdQgaQjabdUgaRbqabaaaleaacqaIYaGmaaaaaa@3531@	Gene, chip and biological sample specific noise *b*_*ijk *_is drawn from zero mean Gaussian distribution *N *(0, σbijk2 MathType@MTEF@5@5@+=feaafiart1ev1aaatCvAUfKttLearuWrP9MDH5MBPbIqV92AaeXatLxBI9gBaebbnrfifHhDYfgasaacH8akY=wiFfYdH8Gipec8Eeeu0xXdbba9frFj0=OqFfea0dXdd9vqai=hGuQ8kuc9pgc9s8qqaq=dirpe0xb9q8qiLsFr0=vr0=vr0dc8meaabaqaciaacaGaaeqabaqabeGadaaakeaaiiGacqWFdpWCdaqhaaWcbaGaemOyai2aaSbaaWqaaiabdMgaPjabdQgaQjabdUgaRbqabaaaleaacqaIYaGmaaaaaa@3531@).
**Rocke EM **[8]:	
Model	*y *= *α *+ *xe*^*n *^+ *ε*
σn2 MathType@MTEF@5@5@+=feaafiart1ev1aaatCvAUfKttLearuWrP9MDH5MBPbIqV92AaeXatLxBI9gBaebbnrfifHhDYfgasaacH8akY=wiFfYdH8Gipec8Eeeu0xXdbba9frFj0=OqFfea0dXdd9vqai=hGuQ8kuc9pgc9s8qqaq=dirpe0xb9q8qiLsFr0=vr0=vr0dc8meaabaqaciaacaGaaeqabaqabeGadaaakeaaiiGacqWFdpWCdaqhaaWcbaGaemOBa4gabaGaeGOmaidaaaaa@30FA@	Multiplicative noise *n *is drawn from zero mean Gaussian distribution *N *(0, σn2 MathType@MTEF@5@5@+=feaafiart1ev1aaatCvAUfKttLearuWrP9MDH5MBPbIqV92AaeXatLxBI9gBaebbnrfifHhDYfgasaacH8akY=wiFfYdH8Gipec8Eeeu0xXdbba9frFj0=OqFfea0dXdd9vqai=hGuQ8kuc9pgc9s8qqaq=dirpe0xb9q8qiLsFr0=vr0=vr0dc8meaabaqaciaacaGaaeqabaqabeGadaaakeaaiiGacqWFdpWCdaqhaaWcbaGaemOBa4gabaGaeGOmaidaaaaa@30FA@).
σε2 MathType@MTEF@5@5@+=feaafiart1ev1aaatCvAUfKttLearuWrP9MDH5MBPbIqV92AaeXatLxBI9gBaebbnrfifHhDYfgasaacH8akY=wiFfYdH8Gipec8Eeeu0xXdbba9frFj0=OqFfea0dXdd9vqai=hGuQ8kuc9pgc9s8qqaq=dirpe0xb9q8qiLsFr0=vr0=vr0dc8meaabaqaciaacaGaaeqabaqabeGadaaakeaaiiGacqWFdpWCdaqhaaWcbaGae8xTdugabaGaeGOmaidaaaaa@3137@	Additive independent noise *ε *is drawn from zero mean Gaussian distribution *N *(0, σε2 MathType@MTEF@5@5@+=feaafiart1ev1aaatCvAUfKttLearuWrP9MDH5MBPbIqV92AaeXatLxBI9gBaebbnrfifHhDYfgasaacH8akY=wiFfYdH8Gipec8Eeeu0xXdbba9frFj0=OqFfea0dXdd9vqai=hGuQ8kuc9pgc9s8qqaq=dirpe0xb9q8qiLsFr0=vr0=vr0dc8meaabaqaciaacaGaaeqabaqabeGadaaakeaaiiGacqWFdpWCdaqhaaWcbaGae8xTdugabaGaeGOmaidaaaaa@3137@).
*μ*_*α*_, σα2 MathType@MTEF@5@5@+=feaafiart1ev1aaatCvAUfKttLearuWrP9MDH5MBPbIqV92AaeXatLxBI9gBaebbnrfifHhDYfgasaacH8akY=wiFfYdH8Gipec8Eeeu0xXdbba9frFj0=OqFfea0dXdd9vqai=hGuQ8kuc9pgc9s8qqaq=dirpe0xb9q8qiLsFr0=vr0=vr0dc8meaabaqaciaacaGaaeqabaqabeGadaaakeaaiiGacqWFdpWCdaqhaaWcbaGae8xSdegabaGaeGOmaidaaaaa@312F@	Background noise (bias) *α *is drawn from Gaussian distribution *N *(*μ*_*α*_, σα2 MathType@MTEF@5@5@+=feaafiart1ev1aaatCvAUfKttLearuWrP9MDH5MBPbIqV92AaeXatLxBI9gBaebbnrfifHhDYfgasaacH8akY=wiFfYdH8Gipec8Eeeu0xXdbba9frFj0=OqFfea0dXdd9vqai=hGuQ8kuc9pgc9s8qqaq=dirpe0xb9q8qiLsFr0=vr0=vr0dc8meaabaqaciaacaGaaeqabaqabeGadaaakeaaiiGacqWFdpWCdaqhaaWcbaGae8xSdegabaGaeGOmaidaaaaa@312F@).
**Hein EM **[11]:	
Model	*PM*_*ijkp *_~ *N*(*S*_*ijkp *_+ *H*_*ijkp*_, τjk2 MathType@MTEF@5@5@+=feaafiart1ev1aaatCvAUfKttLearuWrP9MDH5MBPbIqV92AaeXatLxBI9gBaebbnrfifHhDYfgasaacH8akY=wiFfYdH8Gipec8Eeeu0xXdbba9frFj0=OqFfea0dXdd9vqai=hGuQ8kuc9pgc9s8qqaq=dirpe0xb9q8qiLsFr0=vr0=vr0dc8meaabaqaciaacaGaaeqabaqabeGadaaakeaaiiGacqWFepaDdaqhaaWcbaGaemOAaOMaem4AaSgabaGaeGOmaidaaaaa@3253@), *MM*_*ijkp *_~ *N *(*φS*_*ijkp *_+ *H*_*ijkp*_, τjk2 MathType@MTEF@5@5@+=feaafiart1ev1aaatCvAUfKttLearuWrP9MDH5MBPbIqV92AaeXatLxBI9gBaebbnrfifHhDYfgasaacH8akY=wiFfYdH8Gipec8Eeeu0xXdbba9frFj0=OqFfea0dXdd9vqai=hGuQ8kuc9pgc9s8qqaq=dirpe0xb9q8qiLsFr0=vr0=vr0dc8meaabaqaciaacaGaaeqabaqabeGadaaakeaaiiGacqWFepaDdaqhaaWcbaGaemOAaOMaem4AaSgabaGaeGOmaidaaaaa@3253@), where *PM *refers to perfect match and *MM *to mismatch probe.
*a*_*k*_, bk2 MathType@MTEF@5@5@+=feaafiart1ev1aaatCvAUfKttLearuWrP9MDH5MBPbIqV92AaeXatLxBI9gBaebbnrfifHhDYfgasaacH8akY=wiFfYdH8Gipec8Eeeu0xXdbba9frFj0=OqFfea0dXdd9vqai=hGuQ8kuc9pgc9s8qqaq=dirpe0xb9q8qiLsFr0=vr0=vr0dc8meaabaqaciaacaGaaeqabaqabeGadaaakeaacqWGIbGydaqhaaWcbaGaem4AaSgabaGaeGOmaidaaaaa@3077@	True expression signal log(*S*_*ijkp *_+ 1) is drawn from truncated (realization always ≥ 0) Gaussian distribution *TN *(*x*, σik2 MathType@MTEF@5@5@+=feaafiart1ev1aaatCvAUfKttLearuWrP9MDH5MBPbIqV92AaeXatLxBI9gBaebbnrfifHhDYfgasaacH8akY=wiFfYdH8Gipec8Eeeu0xXdbba9frFj0=OqFfea0dXdd9vqai=hGuQ8kuc9pgc9s8qqaq=dirpe0xb9q8qiLsFr0=vr0=vr0dc8meaabaqaciaacaGaaeqabaqabeGadaaakeaaiiGacqWFdpWCdaqhaaWcbaGaemyAaKMaem4AaSgabaGaeGOmaidaaaaa@324F@), where variance σik2 MathType@MTEF@5@5@+=feaafiart1ev1aaatCvAUfKttLearuWrP9MDH5MBPbIqV92AaeXatLxBI9gBaebbnrfifHhDYfgasaacH8akY=wiFfYdH8Gipec8Eeeu0xXdbba9frFj0=OqFfea0dXdd9vqai=hGuQ8kuc9pgc9s8qqaq=dirpe0xb9q8qiLsFr0=vr0=vr0dc8meaabaqaciaacaGaaeqabaqabeGadaaakeaaiiGacqWFdpWCdaqhaaWcbaGaemyAaKMaem4AaSgabaGaeGOmaidaaaaa@324F@ is drawn from Gaussian distribution *N *(*a*_*k*_, bk2 MathType@MTEF@5@5@+=feaafiart1ev1aaatCvAUfKttLearuWrP9MDH5MBPbIqV92AaeXatLxBI9gBaebbnrfifHhDYfgasaacH8akY=wiFfYdH8Gipec8Eeeu0xXdbba9frFj0=OqFfea0dXdd9vqai=hGuQ8kuc9pgc9s8qqaq=dirpe0xb9q8qiLsFr0=vr0=vr0dc8meaabaqaciaacaGaaeqabaqabeGadaaakeaacqWGIbGydaqhaaWcbaGaem4AaSgabaGaeGOmaidaaaaa@3077@) and *x *is the underlying expression value.
*μ*_*λ*_, σλ2 MathType@MTEF@5@5@+=feaafiart1ev1aaatCvAUfKttLearuWrP9MDH5MBPbIqV92AaeXatLxBI9gBaebbnrfifHhDYfgasaacH8akY=wiFfYdH8Gipec8Eeeu0xXdbba9frFj0=OqFfea0dXdd9vqai=hGuQ8kuc9pgc9s8qqaq=dirpe0xb9q8qiLsFr0=vr0=vr0dc8meaabaqaciaacaGaaeqabaqabeGadaaakeaaiiGacqWFdpWCdaqhaaWcbaGae83UdWgabaGaeGOmaidaaaaa@3144@, *α*_*η*_, *β*_*η*_	Hybridization error term log(*H*_*ijkp *_+ 1) is drawn from truncated Gaussian distribution *TN *(*λ*_*jk*_, ηjk2 MathType@MTEF@5@5@+=feaafiart1ev1aaatCvAUfKttLearuWrP9MDH5MBPbIqV92AaeXatLxBI9gBaebbnrfifHhDYfgasaacH8akY=wiFfYdH8Gipec8Eeeu0xXdbba9frFj0=OqFfea0dXdd9vqai=hGuQ8kuc9pgc9s8qqaq=dirpe0xb9q8qiLsFr0=vr0=vr0dc8meaabaqaciaacaGaaeqabaqabeGadaaakeaaiiGacqWF3oaAdaqhaaWcbaGaemOAaOMaem4AaSgabaGaeGOmaidaaaaa@323A@). Parameter *λ*_*jk *_is drawn from Gaussian distribution *N *(*μ*_*λ*_, σλ2 MathType@MTEF@5@5@+=feaafiart1ev1aaatCvAUfKttLearuWrP9MDH5MBPbIqV92AaeXatLxBI9gBaebbnrfifHhDYfgasaacH8akY=wiFfYdH8Gipec8Eeeu0xXdbba9frFj0=OqFfea0dXdd9vqai=hGuQ8kuc9pgc9s8qqaq=dirpe0xb9q8qiLsFr0=vr0=vr0dc8meaabaqaciaacaGaaeqabaqabeGadaaakeaaiiGacqWFdpWCdaqhaaWcbaGae83UdWgabaGaeGOmaidaaaaa@3144@) and ηjk2 MathType@MTEF@5@5@+=feaafiart1ev1aaatCvAUfKttLearuWrP9MDH5MBPbIqV92AaeXatLxBI9gBaebbnrfifHhDYfgasaacH8akY=wiFfYdH8Gipec8Eeeu0xXdbba9frFj0=OqFfea0dXdd9vqai=hGuQ8kuc9pgc9s8qqaq=dirpe0xb9q8qiLsFr0=vr0=vr0dc8meaabaqaciaacaGaaeqabaqabeGadaaakeaaiiGacqWF3oaAdaqhaaWcbaGaemOAaOMaem4AaSgabaGaeGOmaidaaaaa@323A@ is drawn from gamma distribution Γ^-1^(*α*_*τ*_, *β*_*τ*_).
*α*_*τ*_, *β*_*τ*_	Variance τjk2 MathType@MTEF@5@5@+=feaafiart1ev1aaatCvAUfKttLearuWrP9MDH5MBPbIqV92AaeXatLxBI9gBaebbnrfifHhDYfgasaacH8akY=wiFfYdH8Gipec8Eeeu0xXdbba9frFj0=OqFfea0dXdd9vqai=hGuQ8kuc9pgc9s8qqaq=dirpe0xb9q8qiLsFr0=vr0=vr0dc8meaabaqaciaacaGaaeqabaqabeGadaaakeaaiiGacqWFepaDdaqhaaWcbaGaemOAaOMaem4AaSgabaGaeGOmaidaaaaa@3253@ is drawn from gamma distribution Γ^-1^(*α*_*τ*_, *β*_*τ*_).
*φ*	Fractional binding *φ *can be selected from interval [0, 1].

**Table 6:** Input data requirements. Requirements for the simulator input data used in microarray simulation.

data	Expression values or ratios measured for probes (genes). One value for each time instant per probe is required.
time	Time instants when the expression values are obtained.
genes	Names of the probes.
spot	Location of each probe on the slide (x and y coordinate).
name	Name of the dataset.
type	Type of the input data i.e. cDNA or oligonucleotide expression or ratios.
scale	Scale of the input data, i.e. log or linear scale.

**Table 7:** Segmentation results. Correlation coefficients between the estimated spot intensities and the input data. Histogram segmentation gives the highest correlation with the reference data. All methods give poorer correlations as the image quality is degraded.

Algorithm	Results for image 1	Results for image 2	Results for image 3
FC	0.9952	0.9112	0.8452
HST	0.9962	0.9860	0.9432
SRG	0.9876	0.9602	0.8680
