# Supplementary material for: Simulation of microarray data with realistic characteristics
Source: BMC Bioinformatics. 2006 Jul 18;7:349. doi: 10.1186/1471-2105-7-349 (PMC1574357; doi:10.1186/1471-2105-7-349)
Supplement: Additional file 1 — Microarray simulation model. Matlab implementation of the microarray simulation model. [file 1471-2105-7-349-S1.gz › mamodel_20060511/documentation/microarrayreader.pdf]

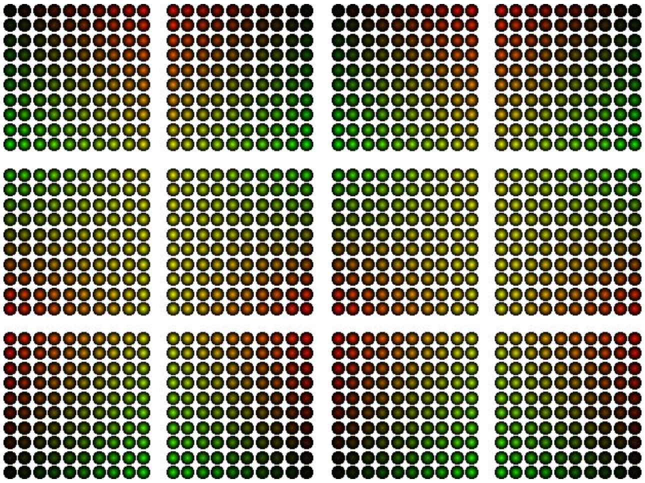

Microarray image reader

Final Report

Raija Lehto 165996
Juho Lahti 167580

Version 1.0, 21.2.2004

raija.lehto <at> tut.fi
lahti25 <at> cs.tut.fi

Version history

**8.12.2003**	**Raija Lehto :**	**The creation of the document**
**8.12.2003**	**Raija Lehto:**	**Introduction and abstract added**
**8.12.2003**	**Raija Lehto:**	**Summary of the project**
**14.2.2004**	**Raija Lehto:**	**Documentation**
**21.2.2004**	**Raija Lehto:**	**Changes made**

Expected credit units: 3 cu

Signature and date:

=====================	===================
Matti Nykter	Marja-Leena Linne
Researcher	Senior Researcher

1. Abstract

Biological measurement systems produce a huge amount of data. One example of biological measurement systems is microarray technology. The microarray is an orderly arrangement of miniaturized test sides on a solid substrate that performs hundreds or thousands of tests simultaneously. In order to make comparisons, cDNA-chips include different states colored with the two fluorodescent substances radiating with different frequencies. Most often in the case of microarray images the data is in the form of a digital (RGB) image and therefore image processing methods are used to automatically analyze the data. It becomes easier to read this information if grids are used.[1-2]

The main purpose of this project is first to design and implement a method for grid alignment. The second phase is to compute an intensity number for each spot and its local background using grid alignment information. The program contains several subareas, metagrid separation, subgrid separation grid alignment for the subgrids and finally the spot localization and intensity value calculations. The system is designed to be flexible and modular in the way that different program components can be easily replaced.

The project group contains two members. The timetable allocation for each project member was planned and the tasks to be done were planned on a weekly basis. These can be viewed in Chapter 2. The timetable worked as planned except for the grid alignment algorithm part, where some delays and changes were noticed.

The project was finished in four and a half months.

Table of Contents

1.ABSTRACT 3

1.INTRODUCTION 6

2.SUMMARY OF THE PROJECT 7

2.1Project background..7
2.2Project objectives, deliverables, planned timetable..7
2.2.1 Requirements..7
2.2.2 Project group objectives..7
2.2.3 Deliverables..7
2.2.4 Planned Timetable..8
2.3Project Organization..8

3.REALIZED PROJECT IMPLEMENTATION..9

3.1 Implementation steps..9
3.1.1 The grid alignment algorithm 9
..10
3.1.2 The spot reader 11
3.1.3 Meetings, week reports, inspections..11
3.2 Deliverables 11
3.3 Problems, delays, changes in project organization and plans..11

4.PROJECT RESULTS AND CONCLUSION..12

5.REFERENCES..13

6. APPENDICES..13

6.1 Examples of commented codes..13
6.3.1 Metagrid..13
6.3.2 The Spot Reader Main..17
6.3.3 The Spot Segmentation..20

Abbreviations and definitions

RGB image	m-by-n-by-3 data array that defines red, green and blue color components in each individual pixel. [3]
Microarray	an orderly arrangement of miniaturized test sides on a solid substrate that performs hundreds or thousands of tests simultaneously. [4]
Green spots	indicate genes "subexpressed" in only one treatment. [5]
Red spots	genes expressed in only one treatment. [5]
Yellow spots	equally expressed genes in both. [5]

1. Introduction

The microarray technique is used in biological samples to measure in parallel e.g. acid sequences or gene expression experiments. The microarray is an orderly arrangement of miniaturized test sides on a solid substrate that performs hundreds or thousands of tests simultaneously. In order to make comparisons, cDNA-chips include different states colored with the two fluorodescent substances radiating with different frequencies. Each spot in an array contains one fluorescently-labelled DNA sequence of a gene and clones of that specific sequence, i.e., one spot represents the measured character of one gene.[1-2]

In the case of microarray images, the data is most often in the form of a digital (RGB) image. Automatic reading of the data from the image is essential in order to be able to utilize the data. The information is usually read by using laser scanning microscopes and there has been research to apply some image processing methods to microarrays.[1-2]

The red and green intensity and the local background have to be read from each spot. It becomes easier to read this information if grids are used [1-2].

In this project we have created a free grid alignment and free spot reader software.

We describe the background of the work including the technology needed. After that we introduce the project organization including the project group members and the client. Next, the project objectives, deliverables and the time allocation plan are described.
Furthermore, the realized project implementation is described including the algorithm descriptions.

2. Summary of the Project

2.1 Project background

The project is carried out at Tampere University of Technology during the academic year 2003-2004 by Juho Lahti and Raija Lehto for a signal processing project course.

The grid alignment systems existing in the market today are mostly commercial products, and therefore there is a need for free grid alignment software.

Some image processing methods are applied for grid alignment and for the spot reader.

2.2 Project objectives, deliverables, planned timetable

The project objectives are to meet the requirements as well as possible. Due to the nature of the images, especially the biological ones, there are always some cases were the algorithms are not especially good.

2.2.1 Requirements

Automatic grid alignment should be robust against noise, contaminations of the slide surface, rotation of grid axes relative to the image, as well as differences and random shifts in spot periodity. Spot intensity estimation should be able to handle different shapes and sizes of spots. There should also be a way of finding the grid semi-automatically if the automatic grid alignment fails.

2.2.2 Project group objectives

1. To implement a fast and robust grid alignment algorithm.
2. To implement an expandable segmentation algorithm.
3. To complete the project before the end of January.

2.2.3 Deliverables

The deliverables are a modular and free matlab code implementation of the microarray reader and the user documentation of the code including algorithm descriptions.

2.2.4 Planned Timetable

Research	Planning	Documentation	Implementation	Testing	All
30h	60h	60h	60h	30h	240h

	September	October	November	December	January	All
Juho Lahti	10h	30h	10h	10h	60h	120h
Raija Lehto	5h	10h	65h	20h	20h	120h

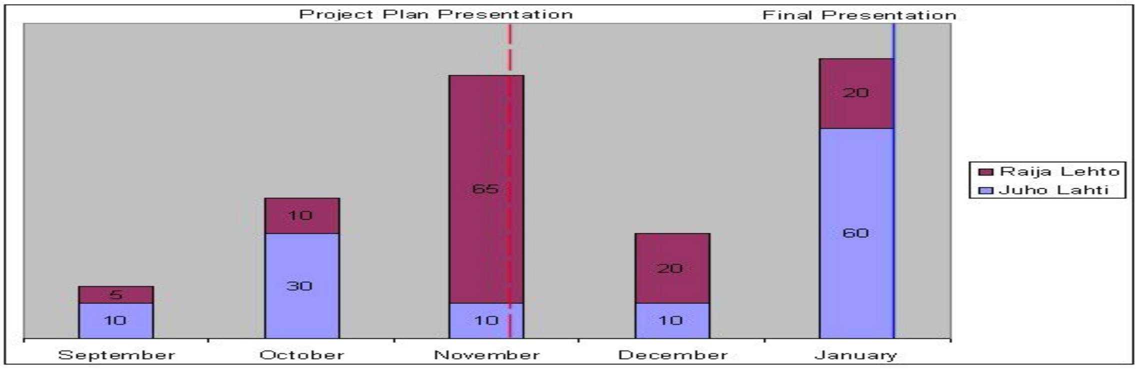

Fig.2: Time usage plan in hours for both project members. We have planned to give a public project plan presentation on 21.11.2003 and final presentation on 30.01.2004. Note that these dates may change.

2.3 Project Organization

There are two project members, Raija Lehto and Juho Lahti. The project manager is Raija Lehto.

The client is researcher Matti Nykter, matti.nykter <at> tut.fi

3. Realized Project Implementation

The project work is realized very much as planned.

3.1 Implementation steps

The grid alignment consists of four main steps, finding the metagrid, finding the grid, drawing the grid, and easygrid. The spot reader consists of two main steps, which are the segmentation and the calculation of the index values. The segmentation step is divided into two main parts.

3.1.1 The grid alignment algorithm

The grid alignment consists of five main steps, finding the metagrid, finding the grid, drawing the grid, easygrid and the editgrid.

1. The first step is to find the metagrid, which includes the metagrid filter that separates grids from the background using projections. First we look for the local minimums from the filtered projections. After that we take the median value of the local minimums. Finally we draw the grids in order to separate the metagrid from the disturbing background borders.
2. The second step is to find places where to draw the grid alignment borders. First, we filter the grid projection to make it easier to find the local maximums from the projections. Second, we select the best of them using the correlation with an ideal grid. Finally, we are able to add the grid borders and refine the placement by using the lowest intensity value under it between two maximums. Figure 3.1 and 3.2 illustrates the above procedure.
3. The next step is to draw the grid lines using the draw grid routine. We draw a grid, metagrid or subgrid depending on parameter stype. We are able to refine the subgrids by the easygrid routine.
4. The easygrid routine computes the row and column intersection points and converts grid data for the spot segmentation part.
5. Finally, the editgrid routine is used to modify the grid placement by hand. It consists of two parts: metagrid and subgrid editor.

The metagrid editor can be used to change the rectangular placement of the grids. The subgrid editor is used to change place of the individual lines by clicking and pulling them to new locations.

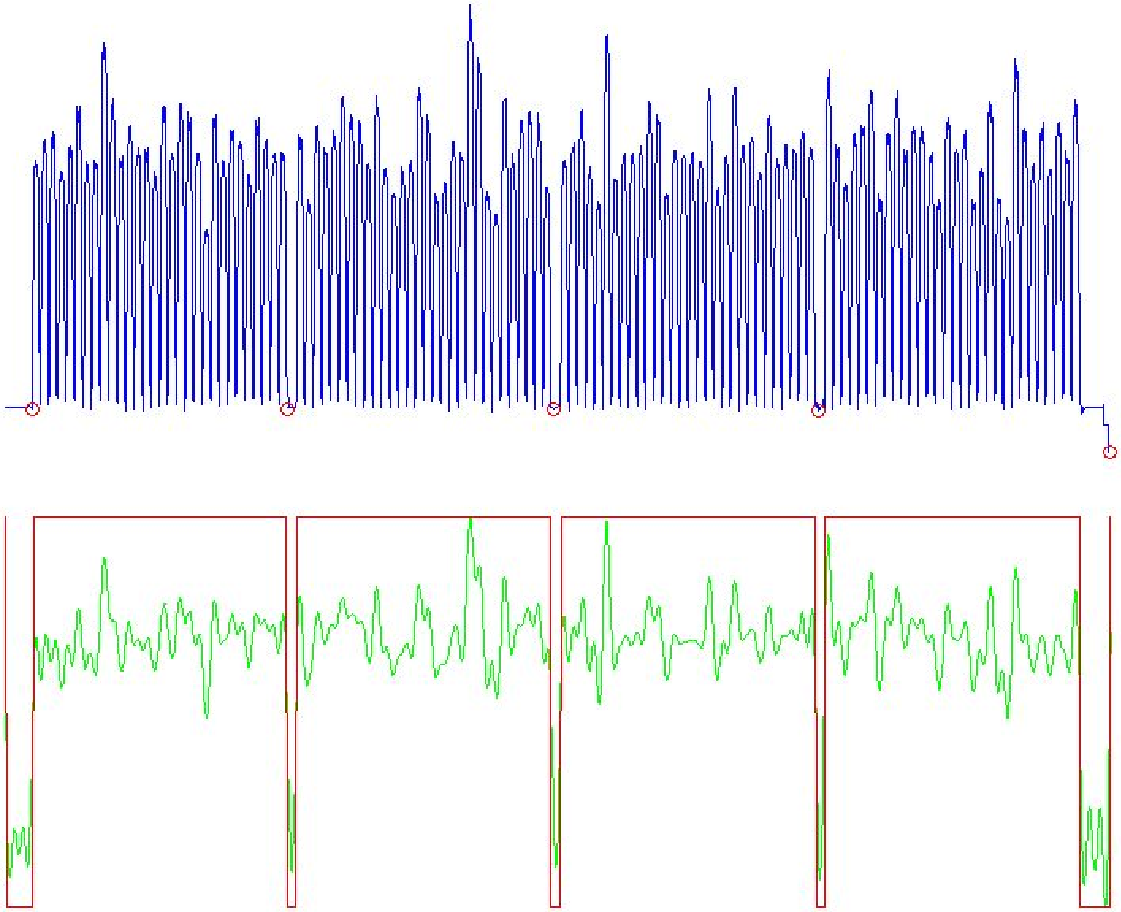

Figure 3.1 Metagrid. The blue line is the original signal. The green line is the filtered signal. The red circles are local minima.

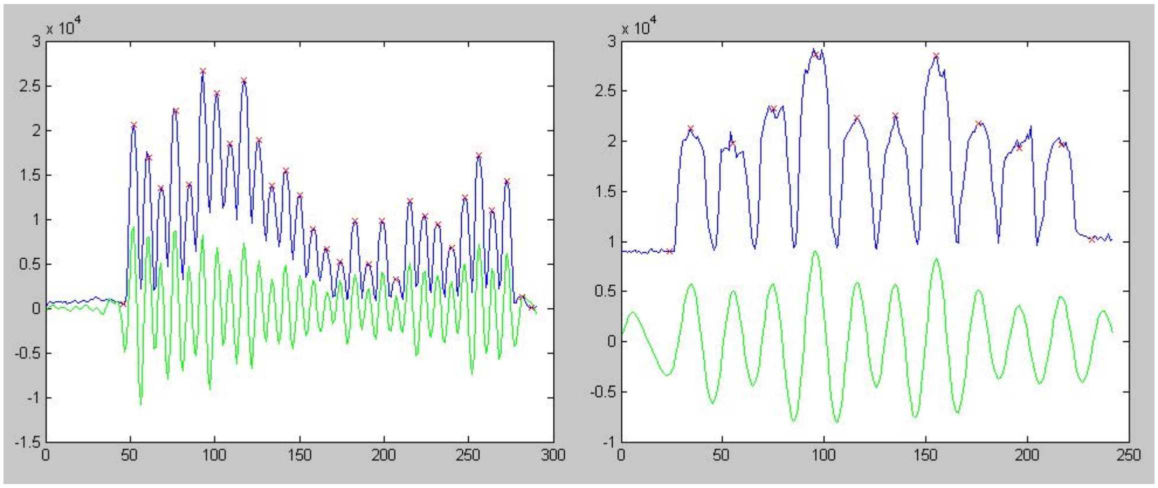

Figure 3.2 Grid. The blue line is the original signal. The green line is the filtered signal. The red dcircles are local maxima.

3.1.2 The spot reader

The spot reader is done in two main steps, which are the segmentation and the calculation of the index values. The segmentation is divided into two parts. The first part is gradient calculation in order to determine the edges of the spot. The second part is to extract the spot from the background.

There are several index values implemented. The index value indicating a spot status in a parameter is called "if-there-is-a-spot-value". The other index values are the maximum likelihood estimation, the median and the minimum and the maximum values. If a spot and the background exist, the index value, "if-there-is-a-spot-value", is set at the difference between the spot median and the background median value. If no spot exists in a space defined by the grids, the variable "if-there-is-a-spot-value" is set at a value minus one as well as all the other spot index values. In the same way, if there is no background, the background index values are set at minus one and "if-there-is-a-spot-value" is set at 3.
Algorithm:
1
2

1. Determine the spot edges with a gradient method, Prewitt
2. Extract the spot from the background
3. Calculate index values for the spot and the background

3.1.3 Meetings, week reports, inspections

There was one meeting with the client on 28.11.2003 about the spot reader and the client approved the approach for the spot reader.

3.2 Deliverables

Matlab code and algorithm descriptions for the client in the middle of the February. The Project plan was delivered to the Course Coordinator in the fall 2003. The final report is delivered to the Course Coordinator during week 8 2004.

3.3 Problems, delays, changes in project organization and plans

The grid alignment took more time than planned. The first part, the metagrid separation from the disturbing background borders, was done by the both group members during the month of January. The whole grid alignment and spot reader system was put together during the first half of February. The delay from the planned schedule was mainly due to the change in the schedule of Juho Lahti, as hours from December were moved to January. The spot reader was accomplished already at the beginning of December as planned. The hours spent were as expected and so was the final implementation result.

4. Project Results and Conclusion

The result is a good microarray image reader and it works as planned.

5. References

1. http://www.cs.tut.fi/~linne/SPP_course/SPP_topics.html
2. Mathiase Katzer, Franz Kummert, Gerhard Sagerer, Robust automatic microarray image analysis, Biefeld University, Faculty of Technology, Germany, April 2002

6. Appendices

6.1 Examples of commented codes

6.3.1 Metagrid

```
function metagrid = findmetagrid(maimage, grids, spots)
% FINDGRID  Find grids from metagrid.
%
%   METAGRID_DATA = FINDMETAGRID(MAIMAGE, GRIDS, SPOTS)
%   finds number of metagrids defined in 2d vector GRIDS.
%   Number of spots is used to find the grids and the
%   number will be stored inside metagrid_data.
%
%   INPUT:
%       GRIDS is a 2 component vector
%           1st component is number of metagrid rows
%           2 nd component is number of metagrid columns
%
%       SPOTS is a 2 component vector
%           1st component is number of spot rows
%           2 nd component is number of spot columns
%
%   OUTPUT:
%       METAGRID_DATA is a cell array of structured
%       data. For more information see GRID_DATA.
%
%   See also FINDMETAGRID, DRAWGRID, ANALYSESPOTS.

   img=sum(maimage,3);
   rows=metagridminimums(double(sum(img,2)), grids(1), spots(1));
   cols=metagridminimums(double(sum(img,1)), grids(2), spots(2));
   metagrid=cell(grids);
   for row=1:grids(1)
      for col=1:grids(2)
         metagrid{row,col} = ...
            struct( 'spots', spots, ...
                  'top', rows(row), 'bottom', rows(row+1), ...
```

```
                'left', cols(col), 'right', cols(col+1) );
        end
    end
% end of findmetagrid

function filtered=metagridfilter(projection,grids,spots);

    % Separate grids from their borders

    N=1000;
    MinFreq=grids/length(projection);
    MaxFreq=grids*spots/length(projection);
    ps=N/2+1;
    b=fir1(N,[MinFreq/3 MaxFreq*1.8],'bandpass',triang(N+1));

    % Next is to handle both projection directions
    % and build up a filter without group delay

    d=size(projection);
    if d(1)==1
        a=zeros(1,ps);
        d=2;
    else
        if d(2)==1
            a=zeros(ps,1);
            d=1;
        end
    end
    s=cat(d,projection-max(projection),a);
    f=filter(b,1,s);
    filtered=f(ps:length(s));

% End of MetagridFilter
function minimums=metagridminimums(projection, grids, spots);

    % Find local minimum areas from a filtered metagrid projection

    filtered=metagridfilter(projection,grids,spots);
    [hole_begs,hole_ends]=minareas(filtered,grids+1);

    minimums=zeros(grids,1);

    % Select median of smallest projection values under minimum areas

    for n=1:length(hole_begs)
        hole=projection(hole_begs(n):hole_ends(n));
        mind=find(hole==min(hole));
        if mod(length(mind),2)==1
```

```
         mind=median(mind);
      else
         mind=median(mind(2:end));
      end
      minimums(n)=mind+hole_begs(n)-1;
   end;

% End of MetagridMinumums

function [hole_begs, hole_ends]=minareas(signal,holes)
% Finds local minimums number of holes from signal
   if length(signal)==0  | holes<1
      hole_begs=[];
      hole_ends=[];
      return;
   end

   %take a global minimum
   [min_value, hole_center]=min(signal);
   hole_beg = hole_center;
   hole_end = hole_center;
   beg_moves=true;
   end_moves=true;

   %expand it (minimum -> hole)
   while( beg_moves | end_moves )
```

```
function [hole_begs, hole_ends]=minareas(signal,holes)
% Finds local minimums number of holes from signal
   if length(signal)==0  | holes<1
      hole_begs=[];
      hole_ends=[];
      return;
   end

   %take a global minimum
   [min_value, hole_center]=min(signal);
   hole_beg = hole_center;
   hole_end = hole_center;
   beg_moves=true;
   end_moves=true;

   %expand it (minimum -> hole)
   while( beg_moves | end_moves )
```

```
      if beg_moves & hole_beg>1
         hole_beg=hole_beg-1;
      end;
      if end_moves & hole_end<length(signal)
        hole_end=hole_end+1;
```

```
    end;
    beg_mean=mean(signal(1:hole_beg));
    end_mean=mean(signal(hole_end:end));
    hole_mean=mean(signal((hole_beg):(hole_end)));
    beg_value=signal(hole_beg);
    end_value=signal(hole_end);
    beg_moves =(abs(beg_value-beg_mean) > abs(beg_value-hole_mean));
    end_moves =(abs(end_value-end_mean) > abs(end_value-hole_mean));
  end;

  %process both sides of the hole recursively
  notspace=length(signal)-(hole_end-hole_beg);
  beg_holes=(holes-.5)*(hole_beg-1)/notspace; %recursion ends when beg_holes<1
  end_holes=(holes-.5)*(length(signal)-hole_end+1)/notspace; % and end_holes<1
  [BB, BE] = minareas(signal(1:(hole_beg-1)),beg_holes);
  [EB, EE] = minareas(signal((hole_end+1):end),end_holes);
  EB=EB + hole_end;
  EE=EE + hole_end;

  %return requested number of holes in order
  hole_begs=[BB hole_beg EB];
  hole_ends=[BE hole_end EE];

% End of MinAreas
```

6.3.2 The Spot Reader Main

```
function[If_SpotR]=spots(maimage,subgrid,channels)
%==========================================================
% function[If_SpotR ]=spots(maimage,subgrid,channels)
%
%  Calculates spot index values for one subgrid.
%  The subgrid is proceeded from left to right for every row.
%
%
%  input:
%          subimage from metagrid  ---- maimage
%          gridpoints:
%               rows    ---- subgrid(:,:,1)
%               columns ---- subgrid(:,:,2)
%         channels   --image colour channels
%  output :
%         contains index value vectors.
%         of one big cell array of them.
%
%
%
%  other functions: segmentation
%                -graythresh --Matlab function, version 6.5.1
%
%==========================================================


 if (nargin==3)
       dims=channels;
else
     dims=2;
end

    rows=size(subgrid,1)
    cols=size(subgrid,2)
    img=im2double(maimage);
    output=cell(rows-1,cols-1,dims); % output

    for row=1:(rows-1),
       for col=1:(cols-1),
           spot=img(subgrid(row,col,1):subgrid(row+1,col,1),  ...
                   subgrid(row,col,2):subgrid(row,col+1,2),:);
           for dim=1:dims
               ch=spot(:,:,dim);
               level=graythresh(ch);
```

```
            [signal, background]=segmentation(ch,'Prewitt',level);
            output{row,col,dim}=Spot_index(signal, background);
         end
      end
   end
   If_SpotR=output;



function [Q]=Spot_index(spot, back)
  %===========================================================
  %  input spot
  %       back --background
  %
  %  output -- different index values
  %   Q=struct('Background_med',Bc_med,'Spot_median',Spot_med,'Spot_Background',
  %   If_Spot,'MLE_background',Bc_MLE,'MLE_spot',Spot_MLE);
  % =============================================
   if ~isempty(spot) & ~isempty(back)

       Bc_med=median(back);
       Spot_med=median(spot);
       If_Spot=Spot_med-Bc_med;
       Bc_Min=min(back);
       Spot_Min=min(spot);
       Bc_Max=max(back);
       Spot_Max=max(spot);
%=============================================
% MLE estimate with 95% confidence intervals
%=============================================
        Bc_MLE=unifit(back);
        Spot_MLE=unifit(spot);

   elseif ~isempty(spot) & isempty(back)
%==============================================
% All spot, no background
%==============================================
       Spot_med=median(spot);
       If_Spot=3;
       Bc_med=-1;
       Spot_MLE=unifit(spot);
       Bc_MLE=-1;
       Bc_Min=-1;
       Spot_Min=min(spot);
       Bc_Max=-1;
       Spot_Max=max(spot);
   elseif isempty(spot) & ~isempty(back)
%==============================================
```

```
% All background, no spot
%==============================================
        Spot_med=-1;
        Bc_med=median(back);
        If_Spot=-1;
 Bc_MLE=unifit(back);
        Spot_MLE=-1;
        Bc_Min=min(back);
        Spot_Min=-1;
        Bc_Max=max(back);
        Spot_Max=-1;
  end
 Q=struct
('Background_med',Bc_med,'Spot_median',Spot_med,'Spot_Background',If_Spot,'MLE_
ba
ckground',Bc_MLE,'MLE_spot',Spot_MLE,'Min_background',Bc_Min,'Max_background
',Bc_Max,'Min_
spot',Spot_Min,'Max_spot',Spot_Max);
```

6.3.3 The Spot Segmentation

```
function [s, bc,spot]=segmentation(R,edgemethod,tresh)
%=====================================================
% function [s, bc,spot]=segmentation(R,edgemethod,tresh)
%
% input :
%    Red, Green or Blue image  -- R
%   'Prewitt' is the only method  -- egdemethod
% output:
%   spot - s (vector)
%   background - bc (vector)
%
%   functions: edges
%              samesize
%
%
%=====================================================
%
%==================================================
[spotreg,x,y]=edges(R,'Prewitt',tresh);

%==============================================
% spot region same size as the original image
%==============================================
spotreg=samesize(spotreg ,R);

%==============================================
% spot segmentation
%==============================================
spot=spotreg.*R;

%==============================================
% extract zeros for spot ID calculations
%==============================================
[i,j,s] = find(spot);


%==============================================
% the background extraction
%==============================================

background=R-spot;

%==============================================
% extract zeros for background ID calculations
```

```
%============================================
[row,col,bc]=find(background);

if isempty(bc)
    [m,n]=size(background);
    bc=zeros(m*n,1);
end
```
