# Supplementary material for: Simulation of microarray data with realistic characteristics
Source: BMC Bioinformatics. 2006 Jul 18;7:349. doi: 10.1186/1471-2105-7-349 (PMC1574357; doi:10.1186/1471-2105-7-349)
Supplement: Additional file 1 — Microarray simulation model. Matlab implementation of the microarray simulation model. [file 1471-2105-7-349-S1.gz › mamodel_20060511/documentation/userguide.pdf]

Microarray simulation model user guide and parameter reference

Matti Nykter
matti.nykter@tut.fi

May 12, 2006

Contents

1 Introduction **2**

2 System requirements **2**

3 Getting started **2**

4 Structure of the model **3**

5 Model parameters **4**

6 Controlling the quality of the simulated data **5**
- 6.1 Noise options 5
- 6.2 Slide options 6
- 6.3 Hybridization options 6
- 6.4 Scanner options 7

7 Simulations in the main paper **7**
- 7.1 Gene knockout experiment 8
- 7.2 Slide simulation 8
- 7.3 Scatter plot example 8
- 7.4 Segmentation example 8

8 Input data **9**

9 Model parameters **11**
- 9.1 General options 12
- 9.2 Noise options 14
  - 9.2.1 Simple error model 15
  - 9.2.2 SNR error model 15
  - 9.2.3 Dror error model 16
  - 9.2.4 Hartemink error model 17
  - 9.2.5 Hierarchical error model 17
  - 9.2.6 Rocke error model 18
  - 9.2.7 Hein error model 19
- 9.3 Slide options 21
- 9.4 Hybridization options 26
- 9.5 Scanner options 30

10 Extending the model **31**
10.1 Handling replicates or PM and MM probes 32
10.2 Adding new error models 32

A License **34**

1 Introduction

This document is a user guide for the microarray simulation model `mamodel` developed at Tampere University of Technology Institute of Signal Processing.

Model is released under the GNU General Public Licence version 2 or later. Copy of the license can be found as an attachment to this document or from `http://www.gnu.org/licenses/licenses.html`.

Latest version of this document is available at `http://www.cs.tut.fi/sgn/csb/mamodel/`. This document is for version 20060307 of the model.

2 System requirements

Model has been implemented using Matlab version 6.5 with signal and image processing toolboxes and statistics toolbox. It may work with older versions of Matlab also and should work with any newer version.

3 Getting started

To get started you need to have a working Matlab installation available. Copy the model from the web site `http://www.cs.tut.fi/sgn/csb/mamodel/`. Extract the downloaded model package. Start Matlab and enter the directory you extracted the model. You can run the model in Matlab with command

```
[output,images,gridimages,meta] = mamodel('gene_deletion.mat');
```

This command will simulate microarray slide using the data loaded from `gene_deletion.mat` and default setting.. This dataset is one of the (simulated) example datasets that are included with the model. Datasets are stored in (and automatically loaded from) directory `datasets/` under the main directory.

The return values are the following. Cell array `output` includes all the information extracted from the slide, cell array `images` contains the simulated images, `gridimages` includes the slide images with grid used to extract

information stored in `output`, and `meta` includes random variable realizations determined during slide generation, e.g. spot locations. Parameters for microarray simulation are read from file `maoptions.m` by default.

You may also specify the options file as a second input parameter

```
[output,images,gridimages,meta] = ...
               mamodel('gene_deletion.mat','optionsfile');
```

This command will read the options from file named `optionsfile.m`. Note that the name of the options file needs to be given without the trailing `.m` (technically this is the name of the function to be called). Options file needs to be the same format as the `maoptions.m` is.

4 Structure of the model

The model is included in following directory structure.

```
mamodel/
 |--datasets/ -- Directory includes all input datasets
 |--mareader/ -- Tools for grid aligment and segmentation
 |--misc/ -- Miscellaneous functions used in model
 |--biologicalnoise.m -- Implementation if biological and
 |                       measurement noise
 |--convertdatatype.m -- Conversion of the input data in
 |                        different data type
 |--hybridization.m -- Hybridization of the input data
 |                      to the slide
 |--hybridizationerrors.m -- Hybridization errors
 |--mamodel.m -- Main file used to run the simulation model
 |--maoptions.m -- Default options file for model
 |--readmadata.m -- Reads input data into the model
 |--segmentslide.m -- Slide segmentation, calls functions
 |                    in mareader directory
 |--slidegen.m -- Generation of a slide
 |--slidegeneration.m -- Function used to control e.g.
 |                        subarray alignment, calls slidegen.m
 |--slidescan.m -- Reads the hybridized slide into the
                   form of a RGB image
```

The order in which the different functions are called is shown in the following tree.

```
mamodel
  |--maoptions (read options)
  |--readmadata (read input data)
  |--convertdatatype (make data type conversions if needed)
  |--biologicalnoise (add biological and measurement noise)
  |--slidegeneration
  |         |--slidegen (generate slide)
  |         |--hybridization (hybridize data)
  |         |--hybridizationerrors (include hyb. errors)
  |
  |--slidescan (scan the slide)
  |--segmentslide (read the information form the slide)
            |--mareader/
```

5 Model parameters

Model parameters are grouped in five groups

General options	These control the general behavior of the model, for example what kind of data is simulated.
Noise options	These are used to determine the statistical properties of the data. These include parameters for introducing population effect, and parameters for several different noise models used to model biological and measurement technology specific noise.
Slide options	These determine the structure of the slide. Also some parameters has an effect to the quality of the slide, specifically the quality of individual spots.
Hybridization options	These determine the quality of the slide. Different types of hybridization errors degrade the quality and add noise to the observed data.
Scanner options	These are used to determine what kind data is obtained from the model. These include parameters that control the saturation and what data is stored in final RGB image

In following sections all these parameters and their purpose are presented in detail.

6 Controlling the quality of the simulated data

Here we introduce how to tune the model parameters. Tables of model parameters are given with possible parameter values. Purpose of these tables is to help user to determine suitable parameter values for a simulation and to give an idea of the range of sensible parameter values. Given parameters are not tuned to correspond to any specific microarray technology, but are chosen to produce results that are typically observed. We have listed three values for each parameter: Good, normal and bad. These parameter values can be used to simulate cDNA microarrays with corresponding quality.

In addition we have included column "Affymetrix" where each parameter that is needed to be set for the simulation of Affymetrix type oligonucleotide microarray is listed. Parameters that are not given a value are not relevant for the simulation of Affymetrix slide, but can be set if desired. These are the parameters for the types of errors that only appear with cDNA microarrays, for example the print tip mark. Same parameter values that are proposed for cDNA microarrays can be used to control the quality of Affymetrix type of arrays also, thus "good" or "bad" parameter values are not separately given for oligonucleotide arrays.

For detailed information about each parameter listed in tables see section 9 or Tables 1 and 2 in the main paper.

6.1 Noise options

With noise options statistical properties of the data can be controlled. Population effect can be applied by defining a kernel that is used to smooth the expression pattern. See parameter documentation for details. In addition the noise model used in simulation needs to be specified and the parameters for noise model defined. It is not trivial to set the noise model parameters such that the obtained data has realistic properties. Methods for selecting the model parameters have been discussed in the publications where the noise models are originally published. Preset noise model parameters are taken from these publications (if available, otherwise determined empirically).

Default parameters for different noise models are the following: Simple noise model (μ, σ^2)=(0.01, 0.001), SNR noise model (μ, SNR)=(0, 10), Dror noise model (μ_{x_i}, $\sigma^2_{x_i}$, μ_f, σ^2_f, α_ϵ, β_ϵ, μ_g, σ^2_g)=(1, 0.01, 0, 36, 13, 0.76, 0, 0.21), Hartemink noise model (μ_{ρ_j}, $\sigma^2_{\rho_j}$, $\sigma^2_{\epsilon_{ij}}$)=(0.2, 0.01, 1), Hierarchical error model (σ^2_ϵ, $\sigma^2_{g_i}$, $\sigma^2_{c_j}$, $\sigma^2_{r_{ij}}$, $\sigma^2_{b_{ijk}}$)=(0.012, 0.010, 0.085, 0.094, 0.011), Rocke noise model (σ^2_n, σ^2_ϵ, μ_α, σ^2_α)=(5, 0.1, 1, 1), Hein noise model (a_k, b^2_k, μ_λ, σ^2_λ, α_η, β_η, α_τ, β_τ)=(0.341, 0.335, 0, 50, 0.5, 1, 0.5, 10).

6.2 Slide options

These are the parameters that has an effect on how the simulated slide looks like.

	Good	Normal	Bad	Affymetrix
S_{type}	cdna	cdna	cdna	oligo
S_{spot}	circle	gaussian	gaussian	
S_{pix}	12	12	12	10
$S_{movprob}$	0.01	0.1	0.5	0.1
S_{mov}	0	1	2	1
S_{μ}	5	5	5	4
S_{σ^2}	0.001	0.01	0.1	0.01
P	0	1	1	
P_p	0.0	0.5	0.9	
P_h	0	3	3	
P_w	0	2	2	
P_b	0	1	2	
C_{prob}	0	0.1	0.25	
C_{num}	0	4	8	
C_{cut}	0	3	6	
B	[4,2]	[4,2]	[4,2]	[1,1]
B_{space}	50	50	50	
B_{curve}	0	1	2	
B_{maxc}	0	3	10	

Parameters N_{slides}, N_{time} should be set to correspond to the number of slides and time points when the slides are made. If simulated data is not from time series, N_{time} should be left empty.

In addition parameters $N_{channels}$, N_{spots}, N_{height}, N_{width}, B_{spots}, B_{height}, B_{width} are set automatically by the model based on the input data. It is possible to set these by hand also. For details see the documentation of each parameter.

6.3 Hybridization options

These parameters control the quality of the slide.

	Good	Normal	Bad	Affymetrix
H_{σ^2}	0.001	0.01	0.1	0.01
H_{errors}	1	1	1	1
$H_{bgnoise}$	10	30	50	20
H_{bgvar}	0.001	0.01	0.03	
H_{bggrad}	1	1	1	1
$H_{noscratch}$	0	1	3	0
$H_{Slength}$	0	0.3	0.9	
H_{Swidth}	0	3	5	
H_{noair}	0	1	3	
$H_{\mu_{air}}$	0	15	30	
$H_{\sigma^2_{air}}$	0	1	10	
H_{bleed}	0	2	10	
$H_{bleedsize}$	0	5	10	
$H_{bleeddist}$	0	0.4	0.4	

Background noise can effectively be controlled using H_{bggrad} parameter. This allows user to set the background noise gradient as a vector. For example $H_{bggrad} = [0, 0.5, 1]$ adds more background noise to the right side of the slide. For more information, see the parameter documentation.

6.4 Scanner options

These are the setting for the virtual scanner used.

	Good	Normal	Bad	Affymetrix
R_{power}	1	10	20	
R_b	16	16	10	
R_{eq}	0	0	0	
R_{th}	7	5	3	
R_{Rch}	2	2	2	
R_{Gch}	1	1	1	
R_{errors}	0	1	1	
R_{angle}	0	0.1	1	
R_{mm}	0	0	1	

7 Simulations in the main paper

Here we discuss the simulations that are shown in the main paper. Relevant parameter values for each simulation are given. Those parameters that are not discussed were set to “normal” as listed above. Noise models where also run using default parameters unless stated otherwise.

7.1 Gene knockout experiment

In this example biological and measurement technology specific noise was added using hierarchical error model with parameters ($\sigma^2_{g_i}$, $\sigma^2_{c_j}$, $\sigma^2_{r_{ij}}$, $\sigma^2_{b_{ijk}}$, σ^2_{ϵ})=(0.01, 0.01, 0.01, 0.01, 0.1). At this stage other sources of errors where not applied, thus all noise visible in the simulated gene expression profiles is due to the hierarchical error model.

7.2 Slide simulation

Relevant parameters for the simulation of the two slides shown in the paper are the following (other parameters are set "normal").

Parameter name	Slide 1	Slide 2
N_{time}	0.05	1
E_{model}	hem	hem
H_{σ^2}	0.001	0.002
$H_{bgnoise}$	30	40
H_{bgvar}	0.01	0.01
$H_{noscratch}$	0	3
H_{curve}	0	2

Noise model parameters where set to ($\sigma^2_{g_i}$, $\sigma^2_{c_j}$, $\sigma^2_{r_{ij}}$, $\sigma^2_{b_{ijk}}$, σ^2_{ϵ})=(0.001, 0.001, 0.001, 0.001, 0.001) in both cases. As stated in the paper, this was done to include very small amount of noise so the spread of the gene knock out would be evident.

7.3 Scatter plot example

Biological and measurement noise were added using hierarchical error model with parameters ($\sigma^2_{g_i}$, $\sigma^2_{c_j}$, $\sigma^2_{r_{ij}}$, $\sigma^2_{b_{ijk}}$, σ^2_{ϵ})=(0.012, 0.010, 0.085, 0.094, 0.011). Slides were generated using "normal" settings.

7.4 Segmentation example

In this example, shown in the main paper, we are interested to study how the quality of the spots effect the segmentation. For this purpose we simulated three microarray slides with different noise parameters. Slides are denoted as (a) high quality slide, (b) noisy slide, (c) disturbing noise over slide. Relevant parameters that were used to obtain the desired effect are summarized in following table

Parameter name	High quality	Noisy slide	Disturbing noise
S_{pix}	12	12	12
S_{spot}	circle	circle	circle
$S_{movprob}$	0	0	0
S_{μ}	4	4	4
S_{σ^2}	0.001	0.005	0.01
P	0	1	1
P_p	-	0.2	0.3
C_{prob}	0	3	4
C_{num}	1	4	4
C_{cut}	0.01	0.1	0.15
H_{σ^2}	0.01	0.02	0.03
$H_{bgnoise}$	10	20	40
H_{bgvar}	0.01	0.02	0.03
$H_{noscratch}$	0	1	3
$H_{Slength}$	-	0.8	0.8
H_{Swidth}	-	5	5
H_{noair}	0	1	3
$H_{\mu_{air}}$	-	10	10
$H_{sigma^2_{air}}$	-	1	1
H_{bleed}	0	1	3
$H_{bleedsize}$	-	5	5
$H_{bleeddist}$	-	0.4	0.4

As a summary, spot size variation was allowed to increase as the quality of the spot degraded as more chords were cut. Also amount of hybridization errors and background noise was increased. Biological noise parameters were not changed as the statistical properties of the data does not have direct effect to the observable slide.

8 Input data

Here we discuss what are the requirements for the input data used in the simulation model. There are seven different parameters that are required for the input data. However, only one input parameter the `data` is required for the model to run. Other parameters, if not defined, are automatically set to a default values. Input data should be saved in the `.mat` file, including the following variables.

Input variable: `data`, type: `cell array`

This is the only mandatory input variable. Data related to each condition is saved into a matrix, each column corresponds to one sample. Different conditions are then stored in cell array. Example: let $\mathbf{x_1}, \ldots, \mathbf{x_n}$ denote the n measurements from normal tissue (condition 1), $\mathbf{x_1}$ is a column vector where each row corresponds to one gene/probe on the microarray. Similarly let $\mathbf{y_1}, \ldots, \mathbf{y_n}$ denote the n measurements from cancer tissue (condition 2). Then matrix $X = [\mathbf{x_1}, \mathbf{x_2}, \ldots, \mathbf{x_n}]$ and $Y = [\mathbf{y_1}, \mathbf{y_2}, \ldots, \mathbf{y_n}]$. Cell array `data` is given as `data` $= \{X, Y\}$.

Input variable: `time`, type: `vector`

Time vector should include the time instants when the different samples are obtained. Thus, the length of this vector needs to be n. Time scale can be in minutes (corresponding e.g. the simulation time scale) or normalized to the interval $[0, 1]$. If `time` is not defined n time points are linearly sampled to the interval $[0, 1]$.

Input variable: `name`, type: `string`

Name of the experiment data set. This is used to identify the data saved on the disk. If not defined `No name` is used as a default value.

Input variable: `info.genes`, type: `cell array`

Names of the genes/probes in the `data`. As noted earlier each row in $\mathbf{x_k}$ corresponds to one gene/probe. Names of these probes are given in this parameter. If there are replicates of the same gene/probe in the dataset, then these replicates needs to have the same name. Names are used to identify the replicates when the error models are applied. `info.genes` is a cell array where each cell is the name of the corresponding probe on the input data, i.e. first cell corresponds to the first row in $\mathbf{x_k}$, second cell to the second row and so one. If this input variable is not defined numbers from 1 to the length of $\mathbf{x_k}$ are used a default names.

If needed gene name information can further be extended. For example the type of the probe can be coded into gene names, e.g. `foobar_PM_1` would mean that gene name is foobar, and it is PM (perfect match) probe from probe set 1. Alternatively and for implementation convenience probe set information could also be stored in in a vector of its own e.g. in `info.probetype`.

Input variable: `info.spots`, type: `matrix`

Locations of the spots on the slide. Coordinates x and y are given for each gene spotted on the data. That is, location for each gene in $\mathbf{x_k}$ needs to be given. In this case coordinates means the absolute order of the spots in integer values e.g. spot with $x = 2$, $y = 3$ is directly above the spot with $x = 2$, $y = 4$. First values in $\mathbf{x}$ and $\mathbf{y}$ corresponds to the first row in $\mathbf{x_k}$, second values to the second row and so on. The `info.spots` is a matrix of form $[\mathbf{x}, \mathbf{y}]$ where $\mathbf{x}$ and $\mathbf{y}$ are column vectors.

Input variable: `info.*`, type: `any`

If needed one can define new fields where to store information related to the input data. It might be of interest to store e.g. information about the probe type. All this information is available for used along with the data in any part of the simulation model. Thus it can be utilized e.g. in error models to identify PM or MM probes and probes that are part of the same probe set.

Input variable: `type`, type: `string`

Type of the input data. Allowed values are `ratios`, `expressions`, and `intensity`. `ratios` refers to gene expression rations. `expressions` is used to refer cDNA type of expression data and `intensity` is used to refer oligonucleotide based expression data.

Input variable: `scale`, type: `string`

Scale of the input data. Possible values are `linear` and `log`. These denote if the input data is in logarithmic or linear scale.

9 Model parameters

Here all the model parameters (from `maoptions.m`) are listed. For each parameter a name (in `maoptions.m` file) and a type of the parameter are given. Also the purpose of each parameter is discussed.

9.1 General options

Parameter name: `opt.outputdatatype`
Parameter symbol: O_{type}
Parameter type: string = {`cdna`, `oligo`, `ratios`}
Description: This parameter determines the type of the simulated microarray slide. possible values are cdna, oligo, and ratios. Note that if the output type is ratios, slide will not be generated but only the error models will be applied to the input data.

Parameter name: `opt.differentialexpressionprobability`
Parameter symbol: O_{dex}
Parameter type: double
Description: This option is used only if input data type is `ratio` and the output type is expressions. This is the percent of genes to be differentially expressed when ratios are transfered to expressions. This is only meant to be used for testing purposes, and thus does not have any real use in simulation of realistic data.

Parameter name: `opt.differentialexpressionmean`
Parameter symbol: $O_{dex\mu}$
Parameter type: double
Description: Mean differential expression in above mentioned ratio to expression transformation.

Parameter name: `opt.differentialexpressionvariance`
Parameter symbol: $O_{dex\sigma}$
Parameter type: double
Description: Variance for above mentioned ratio $\rightarrow$ expression transformation.

Parameter name: `opt.numberofslides`
Parameter symbol: O_{slides}
Parameter type: integer
Description: Number of slides to be generated. Each slide is generated independently.

Parameter name: `opt.sampletimepoints`
Parameter symbol: O_{times}
Parameter type: double vector
Description: Time points when the slides are generated. This need to correspond to the timescale in input data or to be normalized to the interval $[0, 1]$. Length on the `opt.sampletimepoints` needs to equal `opt.numberofslides`. If time series data is not used, then `opt.sampletimepoints` is irrelevant and can be left empty or filled with dummy values.

Parameter name: `opt.interactive`
Parameter symbol: O_{mode}
Parameter type: Boolean
Description: If 1 the grid alignment process is interactive and grid can be aligned manually using mouse.

Parameter name: `opt.segmentimage`
Parameter symbol: O_{seg}
Parameter type: Boolean
Description: If 1 simulated slide image will be segmented, i.e. the spot values are automatically read. This might take a long time for large slides.

Parameter name: `opt.saveidentifier`
Parameter symbol: O_{ident}
Parameter type: string
Description: Identifier for data saved to disk. This string is concatenated to the saved file names.

Parameter name: `opt.savenoisydata`
Parameter symbol: O_{save}
Parameter type: Boolean
Description: If 1 noisy data (i.e. data which the error model is applied, before slide simulation) is saved to disk.

Parameter name: `opt.saveimages`
Parameter symbol: O_{images}
Parameter type: Boolean
Description: If 1, simulated slide images are saved to disk.

Parameter name: `opt.pathforsaveddata`
Parameter symbol: O_{path}
Parameter type: string
Description: Path for the saved data. All the data written to disk is saved in this directory. If not given, current work directory is used.

9.2 Noise options

Parameter name: `noiseopt.nonoise`
Parameter symbol: E
Parameter type: Boolean
Description: If 1 error models are not applied to the input data. Then the data that is used to simulate the slide is free from measurement and biological noise. This might be of interest to bypass error model if real measurement data is used as an input or if one wants to study the e.g. how well the data read from the slide corresponds to input data.

Parameter name: `noiseopt.kernel`
Parameter symbol: E_{kern}
Parameter type: double vector
Description: Kernel used to apply the population effect. This essentially is an impulse response of a (lowpass FIR) filter that is used to smooth the simulated expression pattern. This can be set as a vector including the coefficients of the impulse response.
Motivation: Including population effect is vital when simulating realistic biological data. As simulated data presents data from a single cell, the population effect is needed to get the data that corresponds to a measurement from cell population, which is typically the case with real microarray data.

**Parameter name:**	`noiseopt.numberofcopies`
Parameter symbol:	E_{cp}
Parameter type:	integer
Description:	How many copies of input data are made when the population effect is applied. The population effect is applied again for each copy, i.e. two copies means that for the second copy the population effect is applied twice. Thus, more details are lost. Setting this larger than one can be of interest e.g. when simulating cell cycle dependent data. Then this can be used to model the degradation of population synchrony.
**Parameter name:**	`noiseopt.errormodel`
Parameter symbol:	E_{model}
Parameter type:	string = {`simple`, `snr`, `dror`, `hartemink`, `hem`, `rocke`, `hein`}
Description:	Name of the error model to be used in simulation. Any of the implemented error models can be chosen. See section 10 for details how to add new error models. See the original papers for details on each model. A summary of the error models are also given in main paper, Table 2.

9.2.1 Simple error model

Simple error model adds Gaussian noise to the data.

**Parameter name:**	`noiseopt.model.noisevar`
Parameter symbol:	σ^2
Parameter type:	double
Description:	Variance of the additive Gaussian noise. Noise is drawn from $N(\mu, \sigma^2)$ distribution.
**Parameter name:**	`noiseopt.model.noisemean`
Parameter symbol:	μ
Parameter type:	double
Description:	Mean of the additive Gaussian noise.

9.2.2 SNR error model

SNR error model adds Gaussian noise to the data such that after the noise is added signal-to-noise ratio (SNR) is the predetermined.

**Parameter name:**	`noiseopt.model.noisesnr`
Parameter symbol:	SNR
Parameter type:	double
Description:	Signal-to-noise ratio after the noise is added.
**Parameter name:**	`noiseopt.model.noisemean`
Parameter symbol:	μ
Parameter type:	double
Description:	Mean of the additive Gaussian noise added to the data. Noise is drawn from $N(\mu, \sigma^2)$, where σ^2 is determined based on SNR.

9.2.3 Dror error model

Dror error model is introduced for ratios, computed from Affymetrix data. It is defined as $y = g * (x_i * x) + f + \epsilon$.

**Parameter name:**	`noiseopt.model.drorxibias`
Parameter symbol:	μ_{x_i}
Parameter type:	double
Description:	Binding efficiency of each probe x_i is drawn from Gaussian distribution $N(\mu_{x_i}, \sigma^2_{x_i})$.
**Parameter name:**	`noiseopt.model.drorxivar`
Parameter symbol:	$\sigma^2_{x_i}$
Parameter type:	double
Description:	Variance for binding efficiency.
**Parameter name:**	`noiseopt.model.drorfmean`
Parameter symbol:	μ_f
Parameter type:	double
Description:	Gene specific bias f is drawn from Gaussian distribution $N(\mu_f, \sigma^2_f)$. μ_f is the mean of the distribution.
**Parameter name:**	`noiseopt.model.drorfvar`
Parameter symbol:	σ^2_f
Parameter type:	double
Description:	Variance for gene specific bias from Gaussian distribution.
**Parameter name:**	`noiseopt.model.drorse`
Parameter symbol:	β_ϵ
Parameter type:	double
Description:	Gene and chip specific error ϵ is drawn from Laplace distribution $L(\alpha_\epsilon, \beta_\epsilon)$.

**Parameter name:**	`noiseopt.model.droralphae`
Parameter symbol:	α_ϵ
Parameter type:	double
Description:	Parameter α for gene and chip specific error ϵ drawn from Laplace distribution $L(\alpha_\epsilon, \beta_\epsilon)$.
**Parameter name:**	`noiseopt.model.drorgmean`
Parameter symbol:	μ_g
Parameter type:	double
Description:	Mean of the multiplicative gene and chip specific noise. Noise term g is drawn from log-normal distribution $LN(\mu_g, \sigma_g^2)$.
**Parameter name:**	`noiseopt.model.drorgvar`
Parameter symbol:	σ_g^2
Parameter type:	double
Description:	Variance of the multiplicative gene and chip specific noise from log-normal distribution $LN(\mu_g, \sigma_g^2)$.

9.2.4 Hartemink error model

Hartemink error model is introduced for log ratios, derived from Affymetrix data. It is given in log scale as $y = x + \rho_j + \epsilon_{ij}$.

**Parameter name:**	`noiseopt.model.rhomean`
Parameter symbol:	μ_{ρ_j}
Parameter type:	double
Description:	Mean of the chip specific bias ρ_j, drawn from Gaussian distribution $N(\mu_{\rho_j}, \sigma_{\rho_j}^2)$.
**Parameter name:**	`noiseopt.model.rhovar`
Parameter symbol:	$\sigma_{\rho_j}^2$
Parameter type:	double
Description:	Variance of the chip specific bias ρ_j, drawn from Gaussian distribution $N(\mu_{\rho_j}, \sigma_{\rho_j}^2)$.
**Parameter name:**	`noiseopt.model.sigmarange`
Parameter symbol:	$\sigma_{\epsilon_{ij}}$
Parameter type:	double
Description:	Gene and chip specific error ϵ_{ij} is drawn from Gaussian distribution $N(0, \sigma_{e_{ij}}^2)$.

9.2.5 Hierarchical error model

Hierarchical error model is developed for cDNA data and defined in log scale in form $y = X + \epsilon$, $X = x + g_i + c_j + r_{ij} + b_{ijk}$.

Parameter name: `noiseopt.model.varg`
Parameter symbol: $\sigma^2_{g_i}$
Parameter type: double
Description: $\sigma^2_{g_i}$ is a variance of the gene specific noise g_i drawn from zero mean Gaussian distribution $N(0, \sigma^2_{g_i})$.

Parameter name: `noiseopt.model.varc`
Parameter symbol: $\sigma^2_{c_j}$
Parameter type: double
Description: Variance $\sigma^2_{c_j}$ of the chip specific noise c_j. Noise is drawn from zero mean Gaussian distribution $N(0, \sigma^2_{c_j})$.

Parameter name: `noiseopt.model.varr`
Parameter symbol: $\sigma^2_{r_{ij}}$
Parameter type: double
Description: Variance $\sigma^2_{r_{ij}}$ of the gene and chip specific noise r_{ij}. Noise is drawn from zero mean Gaussian distribution $N(0, \sigma^2_{r_{ij}})$.

Parameter name: `noiseopt.model.varb`
Parameter symbol: $\sigma^2_{b_{ijk}}$
Parameter type: double
Description: Variance $\sigma^2_{b_{ijk}}$ of the gene, chip and biological sample specific noise b_{ijk}. Noise is drawn from zero mean Gaussian distribution $N(0, \sigma^2_{b_{ijk}})$.

Parameter name: `noiseopt.model.vare`
Parameter symbol: σ^2_{ϵ}
Parameter type: double
Description: Variance σ^2_{ϵ} of the independent random noise ϵ. Noise is drawn from zero mean Gaussian distribution $N(0, \sigma^2_{\epsilon})$.

9.2.6 Rocke error model

Rocke error model is developed for cDNA data and is given in form $y = \alpha + xe^n + \epsilon$.

Parameter name: `noiseopt.model.alphamean`
Parameter symbol: μ_α
Parameter type: double
Description: Mean μ_α of the background noise (bias) α. Noise is drawn from Gaussian distribution $N(\mu_\alpha, \sigma^2_\alpha)$.

**Parameter name:**	`noiseopt.model.alphavar`
Parameter symbol:	σ_α^2
Parameter type:	double
Description:	Variance σ_α^2 of the background noise (bias). Noise is drawn from Gaussian distribution $N(\mu_\alpha, \sigma_\alpha^2)$.
**Parameter name:**	`noiseopt.model.sigman`
Parameter symbol:	σ_n^2
Parameter type:	double
Description:	Variance σ_n^2 of the multiplicative proportional noise n. Noise is drawn from zero mean Gaussian distribution $N(0, \sigma_n^2)$.
**Parameter name:**	`noiseopt.model.sigmae`
Parameter symbol:	σ_ϵ^2
Parameter type:	double
Description:	Variance σ_ϵ^2 of the additive independent noise ϵ. Noise is drawn from zero mean Gaussian distribution $N(0, \sigma_\epsilon^2)$.

9.2.7 Hein error model

Hein error model is based on Affymetrix data and includes different noise models for perfect match (PM) and mismatch (MM) probes. Model is defined as $PM_{ijkp} \sim N(S_{ijkp} + H_{ijkp}, \tau_{jk}^2)$, $MM_{ijkp} \sim N(\phi S_{ijkp} + H_{ijkp}, \tau_{jk}^2)$.

**Parameter name:**	`noiseopt.model.amean`
Parameter symbol:	a_k
Parameter type:	double
Description:	True expression signal $\log(S_{ijkp} + 1)$ is drawn from truncated (realization always ≥ 0) Gaussian distribution $TN(x, \sigma_{ik}^2)$, where variance σ_{ik}^2 is drawn from Gaussian distribution $N(a_k, b_k^2)$ and x is the underlying expression value.
**Parameter name:**	`noiseopt.model.bvar`
Parameter symbol:	b_k^2
Parameter type:	double
Description:	Variance b_k^2 is used to determine the true expression signal (see above).

Parameter name: `noiseopt.model.lambdamean`
Parameter symbol: μ_λ
Parameter type: double
Description: Hybridization error term $\log(H_{ijkp} + 1)$ is drawn from truncated Gaussian distribution $TN(\lambda_{jk}, \eta_{jk}^2)$. Parameter λ_{jk} is drawn from Gaussian distribution $N(\mu_\lambda, \sigma_\lambda^2)$.

Parameter name: `noiseopt.model.lambdavar`
Parameter symbol: σ_λ^2
Parameter type: double
Description: Variance σ_λ^2 is used to obtain hybridization error term $\log(H_{ijkp}+1)$, drawn from truncated Gaussian distribution $TN(\lambda_{jk}, \eta_{jk}^2)$. Parameter λ_{jk} is drawn from Gaussian distribution $N(\mu_\lambda, \sigma_\lambda^2)$

Parameter name: `noiseopt.model.etalpha`
Parameter symbol: α_η
Parameter type: double
Description: Hybridization error term $\log(H_{ijkp} + 1)$ is drawn from truncated Gaussian distribution $TN(\lambda_{jk}, \eta_{jk}^2)$. α_η is used to draw η_{jk}^2 from gamma distribution $\Gamma^{-1}(\alpha_\eta, \beta_\eta)$.

Parameter name: `noiseopt.model.etabeta`
Parameter symbol: β_η
Parameter type: double
Description: Hybridization error term $\log(H_{ijkp} + 1)$ is drawn from truncated Gaussian distribution $TN(\lambda_{jk}, \eta_{jk}^2)$. β_η is used to draw η_{jk}^2 from gamma distribution $\Gamma^{-1}(\alpha_\eta, \beta_\eta)$.

Parameter name: `noiseopt.model.taulpha`
Parameter symbol: α_τ
Parameter type: double
Description: α_τ is used to draw variance τ_{jk}^2 from gamma distribution $\Gamma^{-1}(\alpha_\tau, \beta_\tau)$.

Parameter name: `noiseopt.model.taubeta`
Parameter symbol: β_τ
Parameter type: double
Description: β_τ is used to draw variance τ_{jk}^2 from gamma distribution $\Gamma^{-1}(\alpha_\tau, \beta_\tau)$.

9.3 Slide options

**Parameter name:**	`slideopt.sameslideforallchannels`
Parameter symbol:	S_{ch}
Parameter type:	Boolean
Description:	If 1 same prototype slide is used on both channels, thus the hybridization area of the spots is equal for all channels.
Motivation:	If same slide is used for both channels, this indicates significantly higher quality slide. In practice this means that both dyes bind equally.
**Parameter name:**	`slideopt.p_per_s`
Parameter symbol:	S_{pix}
Parameter type:	integer (pixels)
Description:	Area for one spot i.e. simulated spot needs to fit in to the `slideopt.p_per_s` · `slideopt.p_per_s` pixel area.
**Parameter name:**	`slideopt.spottype`
Parameter symbol:	S_{spot}
Parameter type:	string={`gaussian`, `circle`, `gaussiancircle`, `hyperbolic`}
Description:	Type of the spot used in slide simulation. Gaussian spot is modeled using 2-D Gaussian distribution, as circle is plain circle with constant intensity. Gaussiancircle is a spot, where the tails of the Gaussian distribution are filtered out using circle. Hyperbolic spot used polynomial hyperbolic spot shape.
Motivation:	We have implemented few spot types that we have found relevant. New types of models for spot can easily added, see section 10.

Parameter name: slideopt.spotmovementprob
Parameter symbol: $S_{movprob}$
Parameter type: double
Description: Probability for spot to move from optimal location. This parameter models the possibility for a single spot to move randomly from its designated position (the point where it should have been printed). This does not model systematic drift, it can be introduced using `hybridopt.bincurve` parameter.
Motivation: While it is not that common that there is a random movement in the position of individual spots in real microarrays, this is important for testing e.g. grid alignment and segmentation. Introducing random movement makes it possible to test the robustness of the algorithms.

Parameter name: slideopt.spotmovement
Parameter symbol: S_{mov}
Parameter type: integer (pixels)
Description: If spot is determined to move based on `slideopt.spotmovementprob`, then maximum allowed movement bias from designated location, movement in x and y directions are drawn from uniform distribution $U(-S_{mov}, S_{mov})$.
Motivation: Movement is drawn from uniform distribution to effectively get all size of movements. As movement is conditioned by `slideopt.spotmovementprob`, it makes sense to use uniform distribution instead of Gaussian as many spots do not move at all.

Parameter name: slideopt.spotsize
Parameter symbol: S_{μ}
Parameter type: double
Description: Mean radius of the simulated spot. Spot radius is drawn from $N(S_{\mu}, S_{\sigma^2})$ distribution.
Motivation: Spot size is parameterized as a mean of the distribution so that there can be variation in spot size. If variation in size is not desired, then `slideopt.spotsizevariance` can be set small.

Parameter name: slideopt.spotsizevariance
Parameter symbol: S_{σ^2}
Parameter type: double
Description: Allowed variation (variance) of the spot size.

**Parameter name:**	`slideopt.spotsteepness`
Parameter symbol:	S_{steep}
Parameter type:	double
Description:	Steepness of the spot edge. This only effects the polynomial-hyperbolic spot shape. This parameter, together with `slideopt.spotsizevariance` defines how steep is the spot edge.
**Parameter name:**	`slideopt.useprinttip`
Parameter symbol:	P
Parameter type:	Boolean
Description:	If set to 1, print tips can leave a marks to the slide.
**Parameter name:**	`slideopt.printtipprob`
Parameter symbol:	P_p
Parameter type:	double
Description:	Probability for print tip mark to be visible on the spot.
Motivation:	If print tips are set visible, this can be used to control how often print tip mark appears.
**Parameter name:**	`slideopt.maxprinttipheight`
Parameter symbol:	P_h
Parameter type:	integer (pixels)
Description:	Maximum height of the print tip mark, print tip height is drawn from $U(0, P_h)$ distribution. Print tip is modeled using an ellipse.
Motivation:	Variation in the print tip mark size indication the variation in the printing pressure.
**Parameter name:**	`slideopt.maxprinttipwidth`
Parameter symbol:	P_w
Parameter type:	integer (pixels)
Description:	Maximum width of the print tip mark, print tip width is drawn from $U(0, P_w)$ distribution.
**Parameter name:**	`slideopt.maxprinttipbias`
Parameter symbol:	P_b
Parameter type:	integer (pixels)
Description:	Maximum of how much print tip mark is allowed to drift from spot center. Movement in x-axis P_x and y-axis P_y are drawn from $U(0, P_b)$
Motivation:	Similarly as the spots, also the print tip marks are allowed to move away from spot center.

Parameter name: `slideopt.chordcutpropability`
Parameter symbol: C_{prob}
Parameter type: double
Description: Probability for a spot to suffer from a chord cut. This has a direct implication to the quality of the slide. As more spots are suffering chord cuts, the quality of the slide gets poorer.

Parameter name: `slideopt.maxnumberofchordcuts`
Parameter symbol: C_{num}
Parameter type: integer
Description: Maximum number of chords cut for a spot. Number of chord cuts from individual spot is drawn from $U(0, C_{num})$ distribution. This parameter effect the quality of the spots. More chords are cut, less ideal (round) the spot is.

Parameter name: `slideopt.maxchordcut`
Parameter symbol: C_{cut}
Parameter type: integer (pixels)
Description: Maximum depth of the chord cut, cut depth is drawn from $U(0, C_{cut})$. The deeper the cuts, less ideal is the spot.

Parameter name: `slideopt.order`
Parameter symbol: S_{order}
Parameter type: Boolean
Description: If set to 1 input data is ordered according to the spot location information. In 0 data is hybridized to slide in order which they appear in data file. It may be of interest to set this 0 if the number of spots is large, and thus the ordering might take a long time (then the data needs to be in desired order in input data matrix.) Also if this is 0 it is not possible to leave empty spot on the slide.

Parameter name: `slideopt.usebins`
Parameter symbol: S_{bins}
Parameter type: Boolean
Description: If set to 1 subarrays (bins) are used in slide layout.
Motivation: Subarrays are only used with some cDNA technologies, thus this option should be set to 0 when e.g. Affymetrix type of arrays are simulated.

**Parameter name:**	`slideopt.bins`
Parameter symbol:	B
Parameter type:	integer vector (length 2)
Description:	Subarray layout on the slide i.e. number of (subarray)rows and (subarray)columns. For example [4,2] generates 4 rows of subarrays in 2 columns, thus in total the are 8 individual subarrays in the slide.
**Parameter name:**	`slideopt.binspace`
Parameter symbol:	B_{space}
Parameter type:	integer (pixels)
Description:	Space between individual subarrays on the slide. This set how far different subarrays are printed from each other.
**Parameter name:**	`slideopt.channels`
Parameter symbol:	N_{ch}
Parameter type:	integer
Description:	Number of channels (different conditions/dyes) on the slide. For example, with cDNA microarray there are typically 2 channels (red and green) as with Affymetrix there is only one.
**Parameter name:**	`slideopt.spots`
Parameter symbol:	N_{spots}
Parameter type:	integer
Description:	Total number of spots on the slide. This should correspond to the number of rows in the input data matrix.
**Parameter name:**	`slideopt.slides`
Parameter symbol:	N_{slides}
Parameter type:	integer
Description:	For how many slides there are data for. Note that the number of slides to be simulated is set by `opt.numberofslides`.
**Parameter name:**	`slideopt.height`
Parameter symbol:	N_{height}
Parameter type:	integer
Description:	Number of rows of spots on the slide.
**Parameter name:**	`slideopt.width`
Parameter symbol:	N_{width}
Parameter type:	integer
Description:	Number of columns of spots on the slide.

**Parameter name:**	`slideopt.bin.spots`
Parameter symbol:	B_{spots}
Parameter type:	integer vector
Description:	Number of spots in each subarray. For each subarray a number of spots in corresponding array are given. Thus the `slideopt.bin.spots` is a integer vector of length `slideopt.bins(1)` · `slideopt.bins(2)`. Number of spots are listed in column major order (first all subarrays in first column, then all arrays in second column, etc.).
**Parameter name:**	`slideopt.bin.height`
Parameter symbol:	B_{height}
Parameter type:	integer vector
Description:	Number of rows in subarrays. A vector where a number of spots in each subarray are given. Thus the length of the `slideopt.bin.height` equals `slideopt.bins(1)`.
**Parameter name:**	`slideopt.bin.width`
Parameter symbol:	B_{width}
Parameter type:	integer vector
Description:	Number of columns in subarrays. A vector where a number of spots in each subarray are given. Thus the length of the `slideopt.bin.width` equals `slideopt.bins(2)`.

9.4 Hybridization options

**Parameter name:**	`hybridopt.spotnoisevar`
Parameter symbol:	H_{σ^2}
Parameter type:	double
Description:	Multiplicative Gaussian hybridization noise variance. Hybridization noise is drawn from $N(0, H_{\sigma^2})$. This noise term is applied to the simulated spot, thus it has no effect to the background.
**Parameter name:**	`hybridopt.errors`
Parameter symbol:	H_{errors}
Parameter type:	Boolean
Description:	If set to 1 hybridization errors that degrade the slide quality are included in simulation.

Parameter name: `hybridopt.bgnoisecover`
Parameter symbol: $H_{bgnoise}$
Parameter type: double
Description: Percent of the intensity values covered by the background noise. Thus the mean level of background noise is obtained based on the intensity values. Background noise is drawn from Gaussian distribution. See parameter H_{bggrad} for shaping the noise pattern.
Motivation: Background noise level is parameterized as a percent of the intensity values. This makes it possible the set the desired amount of background noise without taking into account the dynamic range of the input data.

Parameter name: `hybridopt.bgnoisevarratio`
Parameter symbol: H_{bgvar}
Parameter type: double
Description: Background noise variance, relative to background noise mean determined using $H_{bgnoise}$.

Parameter name: `hybridopt.bggradient`
Parameter symbol: H_{bggrad}
Parameter type: double vector
Description: Gradient (noise pattern) for background noise. This can be set to produce arbitrary noise pattern for background. for example setting `hybridopt.bggradient=` [00.51]. Will produce a noise gradient such that there are no noise on the left and a lot noise on the right. Gradient [1] produces evenly spread background pattern.

Parameter name: `hybridopt.bggradientdirection`
Parameter symbol: H_{bgdir}
Parameter type: string={`horizontal`, `vertical`, `both`}
Description: This controls how the gradient pattern, defined by H_{bggrad} is applied i.e if gradient is applied from left to right or from top to bottom or on both directions.

**Parameter name:**	`hybridopt.numberofscratch`
Parameter symbol:	$H_{noscratch}$
Parameter type:	integer
Description:	Number of scratches to appear on the slide.
Motivation:	Careless handling of slides may introduce scratches to the slide surface. Also scratches can effectively used to test error tolerance of e.g. segmentation algorithms.
**Parameter name:**	`hybridopt.scratchlength`
Parameter symbol:	$H_{Slength}$
Parameter type:	double
Description:	Maximum length of the scratch from interval $[0, 1]$ (relative to slide dimensions), scratch length is drawn from $U(0, H_{Slength} \cdot \min\{\texttt{slidewidth}, \texttt{slideheight}\})$.
**Parameter name:**	`hybridopt.scratchwidth`
Parameter symbol:	H_{Swidth}
Parameter type:	integer (pixels)
Description:	Width of the scratch.
**Parameter name:**	`hybridopt.numberofairbubbles`
Parameter symbol:	H_{noair}
Parameter type:	integer
Description:	Number of air bubbles visible on the slide.
Motivation:	Air bubbles sometimes appear due to poor hybridization. These are also effective tools for testing the segmentation reliability.
**Parameter name:**	`hybridopt.airbubblemeanradius`
Parameter symbol:	$H_{\mu_{air}}$
Parameter type:	integer (pixels)
Description:	Mean for the air bubble size. Air bubble size is drawn from $N(\mu_{air}, \sigma_{air})$ distribution.
**Parameter name:**	`hybridopt.airbubblevariance`
Parameter symbol:	$H^2_{\sigma_{air}}$
Parameter type:	double
Description:	Allowed variation (variance) for air bubble size radius.

**Parameter name:**	`hybridopt.percentofspotbleeds`
Parameter symbol:	H_{bleed}
Parameter type:	double
Description:	Percent of spots having dye outside spot area (bleeding). Given as a number from interval $[0, 1]$.
Motivation:	This parameter implements the bleeding effect that causes the dyes to be outside the printed spot area. Bleeding is commonly observed in poor quality microarray data.
**Parameter name:**	`hybridopt.spotbleedsize`
Parameter symbol:	$H_{bleedsize}$
Parameter type:	integer
Description:	Size of the spot bleed (i.e. how many times the spot size). This determines how large area around the spot is affected by the bleeding. Direction where the bleeding happens is determined randomly.
**Parameter name:**	`hybridopt.spotbleeddistance`
Parameter symbol:	$H_{bleeddist}$
Parameter type:	double
Description:	How far from the origin the bleeding goes at one time (bleeding is repeated `hybridopt.spotbleedsize` times). Should be ≤ 0.5 (half of the spot size). This together with previous parameter determines how far the bleeding goes.
**Parameter name:**	`hybridopt.bincurve`
Parameter symbol:	B_{curve}
Parameter type:	double
Description:	Parameter used to control the subarray curving (i.e. systematic drift in spot printing). This affects to all the spots within a sub array. See `slideopt.spotmovementprob` for random individual spot movement. Usage: $\tanh(1 : B_{curve})$, 2 is good value.
Motivation:	Systematic drift in spot alignment is common with traditional cDNA technology if the printing instrument is not of high quality.

Parameter name: `hybridopt.maxbincurvature`
Parameter symbol: B_{maxc}
Parameter type: integer (pixels)
Description: Maximum distance the subarray is allowed to curve, drawn from $U(0, B_{maxc})$. This controls what is the maximum amount of pixels any spot is allowed to move from ideal location due to the subarray curving. Bin curve in normalized to the interval $[0, B_{maxc}]$

9.5 Scanner options

Parameter name: `scanopt.scannerpower`
Parameter symbol: R_{power}
Parameter type: double
Description: This is a power of a virtual scanner. Larger the power, more effectively small intensity values are observed, but the larger values tend to saturate. Scanner power is used for histogram equalization, more power yields brighter image.

Parameter name: `scanopt.bits`
Parameter symbol: R_b
Parameter type: integer
Description: The dynamic range of the scanner. Intensity values are quantized to 2^{R_b} interval.

Parameter name: `scanopt.equalizeimagehistogram`
Parameter symbol: R_{eq}
Parameter type: Boolean
Description: If set 1 the histogram equalization is applied. This makes a non-linear transformation for the intensity values, but at the same time makes the image look better (more bright). As a result large values saturate and small are more effectively observed.

Parameter name: `scanopt.thresholdconstant`
Parameter symbol: R_{th}
Parameter type: double
Description: Threshold control parameter used in quantization, values over the threshold are saturated. That is threshold value (and all values larger than that) equals 2^{R_b}.

**Parameter name:**	`scanopt.rchannel`
Parameter symbol:	R_{Rch}
Parameter type:	integer
Description:	Number of channel that is considered to be red dye. This channel is stored in R channel in RGB image.
**Parameter name:**	`scanopt.gchannel`
Parameter symbol:	R_{Gch}
Parameter type:	integer
Description:	Number of channel that is considered as green dye. This channel is stored in G channel in RGB image.
**Parameter name:**	`scanopt.scannererrors`
Parameter symbol:	R_{errors}
Parameter type:	Boolean
Description:	If set to 1 scanner errors are applied.
**Parameter name:**	`scanopt.scandirectionangle`
Parameter symbol:	R_{angle}
Parameter type:	boolean
Description:	Angle at which the slide is scanned. This causes the slide image to be rotated.
Motivation:	If slide is not placed to scanner carefully, the obtained image may not be aligned exactly vertically / horizontally.
**Parameter name:**	`scanopt.channelmaxmissalignment`
Parameter symbol:	R_{mm}
Parameter type:	integer (pixels)
Description:	Misalignment between red and green channel.
Motivation:	If there is an error in alignment of sensors that are used to detect red and green intensity signals, then there might be miss alignment of color channels.

10 Extending the model

To extend the model by adding new features or error models requires the basic skills in Matlab programming. Different types of features are implemented in corresponding functions, e.g. error models are implemented in `biologicalnoise.m`, artefacts in spot shapes and different spot types in `slidegen.m`, hybridization errors in `hybridizationerrors.m` and so on. Thus, if one wants to add e.g. a new type of spot into the model, it should be done by editing the file `slidegen.m` under the function `generateunit()`.

10.1 Handling replicates or PM and MM probes

Replicated genes and different probe sets can be handled using gene name information, stored in `info.genes` as a handle. If needed also additional fields, for example `info.probetype` can be introduced. Here we discuss how, for example, probe set specific effects can be introduced. For example implementations, see existing error models under `misc/` directory.

First probes from the same probe set needs to be collected together. This can be done by finding all the probes that have the same name in `info.genes`. Next PM and MM probes within the probe set can be grouped based on probe set information in `info.genes` or e.g. in user defined field `info.probetype`. Now we have indexes for all PM and MM probes within one probe set. That is, for all the probes that are related to one gene.

Now probe specific effects can easily be applied jointly for all the probes within the probe set. Similarly gene specific noise can be applied to all replicates of the same gene.

As the model allows user to define new fields of information related to data (fields in `info` structure, e.g. `info.probetype`), all types of errors can easily be introduced. All the data stored in `info` structure at the input data are available to use in error models.

10.2 Adding new error models

All error models are called from the file (function) `biologicalnoise.m`. Example function calls of implemented error models can be found from function `noisemodel()` (around line 60 in `biologicalnoise.m`).

All error models are implemented in `m`-files of their own. For example, the implementation of the hierarchical error model can be found from `misc/hemnoisemodel.m`. You should consult existing error models for example implementations and input/output parameters and how to handle e.g. replicated genes/probes in the error models.

Procedure for implementing the new error model is the following:

1. Write the error model implementation in the `m`-file of its own. Save the file e.g. under the `misc/` directory. Note that for the error model input data is given in 3-D matrix where `(:,:,1)` corresponds to first condition, `(:,:,2)` to second and so on. Each row corresponds to a gene and each column to one time instant / different sample (chip).

2. Add a new function call and `elseif` structure in `biologicalnoise.m` under the function `noidemodel()`.

3. Add the parameters of the error model in `maoptions.m` (or similar) file (with new `elseif` structure).

A License

The GNU General Public License
Version 2, June 1991
Copyright © 1989, 1991 Free Software Foundation, Inc.

51 Franklin Street, Fifth Floor, Boston, MA 02110-1301, USA

Everyone is permitted to copy and distribute verbatim copies of this license document, but changing it is not allowed.

Preamble

The licenses for most software are designed to take away your freedom to share and change it. By contrast, the GNU General Public License is intended to guarantee your freedom to share and change free software—to make sure the software is free for all its users. This General Public License applies to most of the Free Software Foundation's software and to any other program whose authors commit to using it. (Some other Free Software Foundation software is covered by the GNU Library General Public License instead.) You can apply it to your programs, too.

When we speak of free software, we are referring to freedom, not price. Our General Public Licenses are designed to make sure that you have the freedom to distribute copies of free software (and charge for this service if you wish), that you receive source code or can get it if you want it, that you can change the software or use pieces of it in new free programs; and that you know you can do these things.

To protect your rights, we need to make restrictions that forbid anyone to deny you these rights or to ask you to surrender the rights. These restrictions translate to certain responsibilities for you if you distribute copies of the software, or if you modify it.

For example, if you distribute copies of such a program, whether gratis or for a fee, you must give the recipients all the rights that you have. You must make sure that they, too, receive or can get the source code. And you must show them these terms so they know their rights.

We protect your rights with two steps: (1) copyright the software, and (2) offer you this license which gives you legal permission to copy, distribute and/or modify the software.

Also, for each author's protection and ours, we want to make certain that everyone understands that there is no warranty for this free software. If the software is modified by someone else and passed on, we want its recipients to

know that what they have is not the original, so that any problems introduced by others will not reflect on the original authors' reputations.

Finally, any free program is threatened constantly by software patents. We wish to avoid the danger that redistributors of a free program will individually obtain patent licenses, in effect making the program proprietary. To prevent this, we have made it clear that any patent must be licensed for everyone's free use or not licensed at all.

The precise terms and conditions for copying, distribution and modification follow.

Terms and Conditions For Copying, Distribution and Modification

0. This License applies to any program or other work which contains a notice placed by the copyright holder saying it may be distributed under the terms of this General Public License. The "Program", below, refers to any such program or work, and a "work based on the Program" means either the Program or any derivative work under copyright law: that is to say, a work containing the Program or a portion of it, either verbatim or with modifications and/or translated into another language. (Hereinafter, translation is included without limitation in the term "modification".) Each licensee is addressed as "you".

   Activities other than copying, distribution and modification are not covered by this License; they are outside its scope. The act of running the Program is not restricted, and the output from the Program is covered only if its contents constitute a work based on the Program (independent of having been made by running the Program). Whether that is true depends on what the Program does.

1. You may copy and distribute verbatim copies of the Program's source code as you receive it, in any medium, provided that you conspicuously and appropriately publish on each copy an appropriate copyright notice and disclaimer of warranty; keep intact all the notices that refer to this License and to the absence of any warranty; and give any other recipients of the Program a copy of this License along with the Program.

   You may charge a fee for the physical act of transferring a copy, and you may at your option offer warranty protection in exchange for a fee.

2. You may modify your copy or copies of the Program or any portion of it, thus forming a work based on the Program, and copy and distribute such modifications or work under the terms of Section 1 above, provided that you also meet all of these conditions:

(a) You must cause the modified files to carry prominent notices stating that you changed the files and the date of any change.

(b) You must cause any work that you distribute or publish, that in whole or in part contains or is derived from the Program or any part thereof, to be licensed as a whole at no charge to all third parties under the terms of this License.

(c) If the modified program normally reads commands interactively when run, you must cause it, when started running for such interactive use in the most ordinary way, to print or display an announcement including an appropriate copyright notice and a notice that there is no warranty (or else, saying that you provide a warranty) and that users may redistribute the program under these conditions, and telling the user how to view a copy of this License. (Exception: if the Program itself is interactive but does not normally print such an announcement, your work based on the Program is not required to print an announcement.)

These requirements apply to the modified work as a whole. If identifiable sections of that work are not derived from the Program, and can be reasonably considered independent and separate works in themselves, then this License, and its terms, do not apply to those sections when you distribute them as separate works. But when you distribute the same sections as part of a whole which is a work based on the Program, the distribution of the whole must be on the terms of this License, whose permissions for other licensees extend to the entire whole, and thus to each and every part regardless of who wrote it.

Thus, it is not the intent of this section to claim rights or contest your rights to work written entirely by you; rather, the intent is to exercise the right to control the distribution of derivative or collective works based on the Program.

In addition, mere aggregation of another work not based on the Program with the Program (or with a work based on the Program) on a volume of a storage or distribution medium does not bring the other work under the scope of this License.

3. You may copy and distribute the Program (or a work based on it, under Section 2) in object code or executable form under the terms of Sections 1 and 2 above provided that you also do one of the following:

   (a) Accompany it with the complete corresponding machine-readable source code, which must be distributed under the terms of Sec-

tions 1 and 2 above on a medium customarily used for software interchange; or,

(b) Accompany it with a written offer, valid for at least three years, to give any third party, for a charge no more than your cost of physically performing source distribution, a complete machine-readable copy of the corresponding source code, to be distributed under the terms of Sections 1 and 2 above on a medium customarily used for software interchange; or,

(c) Accompany it with the information you received as to the offer to distribute corresponding source code. (This alternative is allowed only for noncommercial distribution and only if you received the program in object code or executable form with such an offer, in accord with Subsection b above.)

The source code for a work means the preferred form of the work for making modifications to it. For an executable work, complete source code means all the source code for all modules it contains, plus any associated interface definition files, plus the scripts used to control compilation and installation of the executable. However, as a special exception, the source code distributed need not include anything that is normally distributed (in either source or binary form) with the major components (compiler, kernel, and so on) of the operating system on which the executable runs, unless that component itself accompanies the executable.

If distribution of executable or object code is made by offering access to copy from a designated place, then offering equivalent access to copy the source code from the same place counts as distribution of the source code, even though third parties are not compelled to copy the source along with the object code.

4. You may not copy, modify, sublicense, or distribute the Program except as expressly provided under this License. Any attempt otherwise to copy, modify, sublicense or distribute the Program is void, and will automatically terminate your rights under this License. However, parties who have received copies, or rights, from you under this License will not have their licenses terminated so long as such parties remain in full compliance.

5. You are not required to accept this License, since you have not signed it. However, nothing else grants you permission to modify or distribute the Program or its derivative works. These actions are prohibited by law if

you do not accept this License. Therefore, by modifying or distributing the Program (or any work based on the Program), you indicate your acceptance of this License to do so, and all its terms and conditions for copying, distributing or modifying the Program or works based on it.

6. Each time you redistribute the Program (or any work based on the Program), the recipient automatically receives a license from the original licensor to copy, distribute or modify the Program subject to these terms and conditions. You may not impose any further restrictions on the recipients' exercise of the rights granted herein. You are not responsible for enforcing compliance by third parties to this License.

7. If, as a consequence of a court judgment or allegation of patent infringement or for any other reason (not limited to patent issues), conditions are imposed on you (whether by court order, agreement or otherwise) that contradict the conditions of this License, they do not excuse you from the conditions of this License. If you cannot distribute so as to satisfy simultaneously your obligations under this License and any other pertinent obligations, then as a consequence you may not distribute the Program at all. For example, if a patent license would not permit royalty-free redistribution of the Program by all those who receive copies directly or indirectly through you, then the only way you could satisfy both it and this License would be to refrain entirely from distribution of the Program.

   If any portion of this section is held invalid or unenforceable under any particular circumstance, the balance of the section is intended to apply and the section as a whole is intended to apply in other circumstances.

   It is not the purpose of this section to induce you to infringe any patents or other property right claims or to contest validity of any such claims; this section has the sole purpose of protecting the integrity of the free software distribution system, which is implemented by public license practices. Many people have made generous contributions to the wide range of software distributed through that system in reliance on consistent application of that system; it is up to the author/donor to decide if he or she is willing to distribute software through any other system and a licensee cannot impose that choice.

   This section is intended to make thoroughly clear what is believed to be a consequence of the rest of this License.

8. If the distribution and/or use of the Program is restricted in certain countries either by patents or by copyrighted interfaces, the original

copyright holder who places the Program under this License may add an explicit geographical distribution limitation excluding those countries, so that distribution is permitted only in or among countries not thus excluded. In such case, this License incorporates the limitation as if written in the body of this License.

9. The Free Software Foundation may publish revised and/or new versions of the General Public License from time to time. Such new versions will be similar in spirit to the present version, but may differ in detail to address new problems or concerns.

   Each version is given a distinguishing version number. If the Program specifies a version number of this License which applies to it and "any later version", you have the option of following the terms and conditions either of that version or of any later version published by the Free Software Foundation. If the Program does not specify a version number of this License, you may choose any version ever published by the Free Software Foundation.

10. If you wish to incorporate parts of the Program into other free programs whose distribution conditions are different, write to the author to ask for permission. For software which is copyrighted by the Free Software Foundation, write to the Free Software Foundation; we sometimes make exceptions for this. Our decision will be guided by the two goals of preserving the free status of all derivatives of our free software and of promoting the sharing and reuse of software generally.

No Warranty

11. Because the program is licensed free of charge, there is no warranty for the program, to the extent permitted by applicable law. Except when otherwise stated in writing the copyright holders and/or other parties provide the program "as is" without warranty of any kind, either expressed or implied, including, but not limited to, the implied warranties of merchantability and fitness for a particular purpose. The entire risk as to the quality and performance of the program is with you. Should the program prove defective, you assume the cost of all necessary servicing, repair or correction.

12. In no event unless required by applicable law or agreed to in writing will any copyright holder, or any other

party who may modify and/or redistribute the program as permitted above, be liable to you for damages, including any general, special, incidental or consequential damages arising out of the use or inability to use the program (including but not limited to loss of data or data being rendered inaccurate or losses sustained by you or third parties or a failure of the program to operate with any other programs), even if such holder or other party has been advised of the possibility of such damages.

End of Terms and Conditions
